# A promising iPS-based single-cell cloning strategy revealing signatures of somatic mutations in heterogeneous normal cells

**DOI:** 10.1016/j.csbj.2020.08.026

**Published:** 2020-09-03

**Authors:** Xuexia Miao, Yueying Li, Caihong Zheng, Lifei Wang, Chen Jin, Lei Chen, Shuangli Mi, Weiwei Zhai, Qian-Fei Wang, Jun Cai

**Affiliations:** a Key Laboratory of Genomic and Precision Medicine, Beijing Institute of Genomics, Chinese Academy of Sciences, China National Center for Bioinformation, Beijing 100101, China; b University of Chinese Academy of Sciences, Beijing 100049, China; c Department of Human Genetics, Genome Institute of Singapore, Agency for Science, Technology and Research, Singapore 138672, Singapore; d Key Laboratory of Zoological Systematics and Evolution, Institute of Zoology, Chinese Academy of Sciences, Beijing, China; e Center for Excellence in Animal Evolution and Genetics, Chinese Academy of Sciences, Kunming, China

**Keywords:** Somatic mutations, Single-cell genomics, Single-cell cloning, Induced pluripotent stem cells (iPSCs)

## Abstract

Single-cell genomics has advanced rapidly as trace-DNA amplification technologies evolved. However, current technologies are subject to a variety of pitfalls such as contamination, uneven genomic coverage, and amplification errors. Even for the “golden” strategy of single stem cell-derived clonal formation, high-fidelity amplification is applicable merely to single stem cells. It’s still challenging to accurately define somatic mutations of a single cell in various cell types. Herein, we provided evidence, for the first time, to prove that induced pluripotent stem cells (iPS cells or iPSC), being a single somatic cell-derived clone, are recording almost identical (>90%) mutational profile of the initial cell progenitor. This finding demonstrates iPS technique, applicable to any cell type, can be utilized as a cell cloning strategy favorable for single-cell genomic amplification. This novel strategy is not limited by cell-type constraints or amplification artifacts, and thus enables our detailed investigation on the characteristics of somatic mutations in heterogeneous normal cells.

## Graphical abstract

**Figure:**
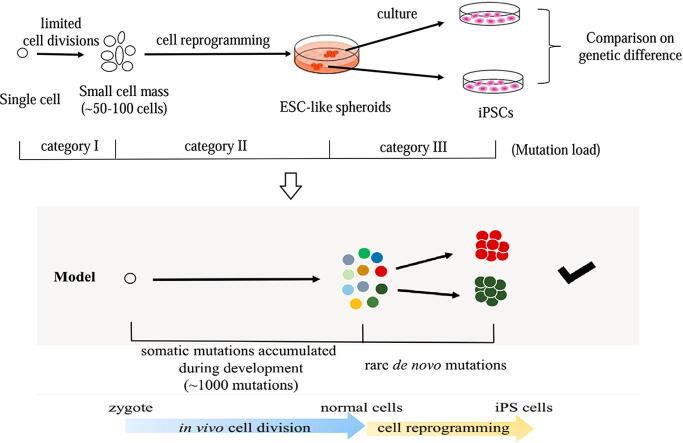

## 1 Introduction

Numerous non-inherited somatic mutations, distinct from those of germ-line origin, occur during DNA replication per cell division and record the unique genetic “history” of each proliferating cell. In previous tumor studies, somatic mutations were of particular concern because some drove the rapid proliferation of abnormal cells and contributed to tumorigenesis [1]. These highlighted driver mutations are present and observable in most cells of a tumor mass owing to the characteristics of tumor clonality. However, clonality is not a standard feature in all types of normal cells [2], [3], where the genetic status of each cell lineage is potentially distinct even within a homogeneous cell type [4], [5], [6]. Therefore, the somatic mutations present in rare or single cells remain to be investigated, especially when they are utilized for tracing the heterogeneity and the dynamic phylogenetic lineages in populations of normal or tumor cells [7].

The somatic mutations in rare cells or even in a single cell have largely remained unexplored via routine deep-sequencing because of their ultra-low frequency hidden in the genetic background of heterogeneous cells. However, the recent development of advanced biotechnologies enables the screening of such somatic mutations [8]. Amplification technology of the single-cell genome is a representative method, which has been implemented in two principal forms. It comprises a straightforward strategy to extract and clone nucleic acids from a single cell using amplification reaction reagents, just as multiple displacement amplification (MDA) or multiple annealing and looping-based amplification cycles (MALBAC), or linear amplification via transposon insertion (LIANTI) did [9], [10], [11]. However, *ex-vivo* molecular cloning beginning from the minimal amount of genetic materials in a single cell inevitably results in DNA contamination, uneven genomic coverage, allele dropout, and amplification error. The above negative factors have been posing problems for accurate estimation on genetic variation profile of a single cell, although some effective improvements through linear DNA amplification, low-temperature cell lysis and correction with the complementary strand were made [3], [12], [13], [14], [15]. Another strategy is cell cloning; that is, to create a clonal cell strain derived from an individual cell, thereby enabling the ancestor cell genome to undergo high-fidelity and full-coverage expansion with the benefit of mitotic cell divisions during cell culture [3], [16]. Although investigators have successfully surveyed somatic mutations utilizing series of cell clones from single stem cells such as embryonic stem and hematopoietic stem cells, it is challenging to establish and maintain single cell clones derived from differentiated cells in multi-cellular organisms. Overall, it is thus generally believed that a technical improvement is required to overcome the existing defects of single-cell genome amplification.

The stem cells that propagate through numerous cycles of cell division possess the property of self-renewal, which contributes to the success of cell cloning from a single stem cell. This provides the reason to suppose that cell cloning might be applicable to distinct lineages of cells as well as stem cells if the cell characteristic of propagation extended to the differentiated somatic cells. Notably, the process of cell reprogramming introduces a practical solution to activate the self-renewal property as well as pluripotency for differentiated somatic cells, although the biochemistry involved in reprogramming the nucleus is not precisely understood. For example, pluripotent cell lines can be established directly from adult cells via somatic cell nuclear transplantation (SCNT) and induced pluripotent stem (iPS) techniques [17], [18]. Subsequently, the question may thus be raised regarding whether cell reprogramming is alternatively available for the cell cloning of various adult cells to amplify the DNA of a single cell for the purpose of screening somatic mutations, albeit at the expense of epigenomic reconfiguration for the cells. To address this issue, we need to prove the validity of two underlying assumptions. The first assumption is that each cell line established via reprogramming is clonally derived from an individual somatic cell. The experimental protocols of cell reprogramming and culture might support this assumption; however, its direct evidence is lacking [19]. The second important assumption is that the genomic profile of the cloned induced pluripotent stem cells (iPS cells or iPSC) accurately characterizes the genomic variations in the original single somatic cell. Through genetic comparisons with the parental cells, researchers have observed many mutations in reprogrammed stem cells [20], [21]; however, the ultimate sources of these observable mutations, e.g., whether they are *de novo* mutations induced during reprogramming or pre-existing in mosaic form in somatic cells, are not definite. Some evidence suggested that at least half or two-thirds of the mutations observed in iPSCs represented the genomic accumulation of somatic mutations in the parental cells, which did not facilitate the acquisition of pluripotency for reprogramming [20], [21], [22], [23]. Other evidence, however, supported the contrary view [24], [25]. Therefore, an effective experimental design is required to evaluate the actual number of *de novo* mutations that actually occur during reprogramming and whether or not the latter assumption is tenable.

In this study, the conclusion that each iPSC line is clonal from an individual somatic cell was inferred through our analysis on mutation frequencies. Furthermore, we proposed a design scheme to obtain a precise estimation on the upper-limit amount of *de novo* mutations in the total observable mutations in iPSCs. The results demonstrate that rare *de novo* mutations are introduced during reprogramming and the genotype of iPSCs is almost identical to that of its initial single cell progenitor. Together, the evidence supports the conclusion that iPS-based cell reprogramming is an effective cell cloning strategy to accurately amplify the genomic information of a single cell, which contributed to our subsequent screening of somatic mutations in heterogeneous cells.

## 2 Material and methods

### 2.1 IPSC induction and cell culture

All animal procedures were performed according to the National Institute of Biological Sciences Guide for the care and use of laboratory animals. neural stem cells (NSCs) were isolated from a newborn all-iPS mouse, which was generated from an iPSC line through tetraploid complementation [26], [27]. Plasmid preparation, and the procedure of iPS derivation were performed according to the methods described previously [26], [28].

Single cells were picked from digested NSCs and plated individually on a 96-well dish with media of DMEM/F12 (Life Technologies) supplemented with 1 × B27 (Life Technologies), 20 ng/ml murine EGF (Peprotech), and 20 ng/ml bFGF (Peprotech). Neurospheres, that were formed approximately 5–6 days later, were then digested and Tet-on induced on the feeder cells with regular embryonic stem cell (ESC) media supplemented with 1 μg/ml doxycycline and 10 ng/ml ascorbic acid. Approximately 15–18 days later, the ESC-like spheroids were mechanically picked up and further cultured into iPSC lines. IPSC culture medium contained DMEM (Life Technologies) supplemented with 15% fetal bovine serum (FBS), 1 mM L-glutamine, 0.1 mM mercaptoethanol, 1% nonessential amino acids, and 1000 U/ml leukocyte inhibitory factor (LIF) (all from Chemicon). Culture dishes were kept at 37 °C in a humidified atmosphere of 5% CO_2_ in air.

### 2.2 Reverse transcription-polymerase chain reaction (RT-PCR) analysis on pluripotency markers

Total RNA was extracted using TRIzol reagent and was converted into cDNA using a Reverse Transcriptase System (A3500, Promega). Polymerase chain reaction (PCR) amplification was carried out for 30 cycles (94 °C, 30 sec; 60 °C, 30 sec; 72 °C, 30 sec). The supplemental information included the primer sequences used for RT-PCR analysis (Supplementary Table S4).

### 2.3 Alkaline phosphatase (AP) staining and immunocytochemical analysis

AP staining was performed using the Leukocyte Alkaline Phosphatase Kit (Sigma-Aldrich) following protocols provided by the manufacturer. For immunofluorescence, colonies were fixed for 2 h at room temperature with 4% paraformaldehyde and then incubated at room temperature for 15 min with 1% Triton X-100/phosphate buffer (PBS). Cells were washed three times in PBS and blocked at 37 °C for over 3 h with 4% normal goat serum (Chemicon). Subsequently, cells were incubated at 4 °C overnight with primary antibody against Oct4 (1:500, Santa Cruz Biotechnology), SSEA-1 (1:500, Chemicon), Nanog (1:500, Cosmobio), or Sox2 (1:500, Abcam). Cells were washed three times in PBS and incubated at 37 °C for 2 h with goat anti-rabbit Alexa-Flour 594-conjugated (Life Technologies) and goat anti-mouse Alexa-Fluor IgG or IgM 633-conjugated (Molecular Probes) secondary antibodies (1:500 in 1% normal goat serum in PBS). Unbound secondary antibody was removed using three washes with PBS. Nuclei were identified by DAPI (Invitrogen) staining at a dilution of 1:1,000,000 at room temperature for 5 min. Images were acquired using a confocal laser scanning microscope (LSM 510 META, Carl Zeiss).

### 2.4 Ex vivo and *in vivo* differentiation for iPSCs

*Ex vivo* differentiation was performed by the embryoid body (EB) formation method. The cells were dissociated into single cells and plated at 2 × 10^5^ cells/ml in suspension culture in the absence of LIF using IMDM (Life Technologies) supplemented with 15% FBS, 1 mM L-glutamine, and 0.1 mM nonessential amino acids with an Ultra-Low Attachment 6-well plate. *In vivo* differentiation utilized the formation of teratomas. Briefly, 2 × 10^6^ cells suspended in 200 µl PBS were injected under the inguinal skin of severe combined immunodeficient (SCID) mice. After 3-4 weeks, the teratomas were excised, fixed in 10% paraformaldehyde, and subjected to histological examination with hematoxylin and eosin staining [29], [30].

### 2.5 Sequencing and somatic single nucleotide variation (SNV) calling

Genomic DNA was extracted from the cell pellets using the DNeasy Mini Kit (Qiagen). Exome and WGS libraries of all the samples were constructed according to the manufacturers’ standard protocols of Illumina Hiseq. A total of 2 × 100 bp or 2 × 150 bp paired-end reads were produced using the Illumina sequencing system. The uniquely alignable reads on mm9 (UCSC) obtained using the Burrows-Wheeler Alignment (BWA) algorithm were retained for downstream analysis [31]. The MuTect algorithms were used to identify candidate somatic SNVs [32]. The following criteria were applied for SNV filtering: (1) variant sites had a minimum coverage of 15 and Phred-scaled base quality above 15; (2) the mutant allele SNV frequency was in the range of 0.3-0.7, whereas it was 0 or 1 in the control sample; (3) the mutant allele was supported by at least two reads in the forward strand and two reads in the reverse strand; (4) sites in dbSNP were additionally excluded.

### 2.6 SNV validation by Sequenom^TM^

Sequenom^TM^ was employed to verify the called SNVs. Random selected primers were designed using the Online Tools in the Sequenom^TM^ Assay Design Suite (https://www.mysequenom.com/Tools). The percentage of mutant alleles was estimated using the default settings of the MassARRAY Typer 4.0 Analyzer. The false positive rate of SNV calling was estimated according to the Sequenom^TM^ validation results.

### 2.7 The copy number aberration (CNA) validation by qPCR

The CNA on chromosome 12 was validated via quantitative polymerase chain reaction (qPCR). Ten groups of primers were designed for this CNA with four non-CNA regions as the “reference” (Supplementary Table S5). The qPCR was performed in samples of iPSCs and NSCs.

### 2.8 Collections of SNV data in various types of somatic cells

The iPSC lines (APC-iPSCs, MEF-iPSCs, and MSC-iPSCs) and corresponding genomic data were collected from our previous study [5], [33]. The somatic SNV data of four individual cells (two from the CD34 + cells, and two from the CD34- cells) were retrieved from the genomic data of four iPSC lines. These iPSCs were derived from the CD34 + cells and the CD34- cells, sorted from the bone marrow mononuclear cells of an adult healthy male [5]. The SNV data observable in the stem-cell clonal HSPCs were downloaded from NCBI public database [16]. All the public genomic data and the relative cell lines were summarized in Table S3.

### 2.9 Replication timing regions, density of DNase I hypersensitive sites (DHS), and density of somatic SNVs

The replication timing profiles were downloaded from ENCODE (https://www.encodeproject.org/). The cell-type matched DNase I hypersensitivity data were downloaded from Epigenomics Roadmap and ENCODE. The mean density of DHS per 10 Mb was calculated as well as the density of somatic SNVs.

## 3 Results

### 3.1 The strategy and its implementation to distinguish the origins of the mutations in iPS cells

**Fig. 1:**
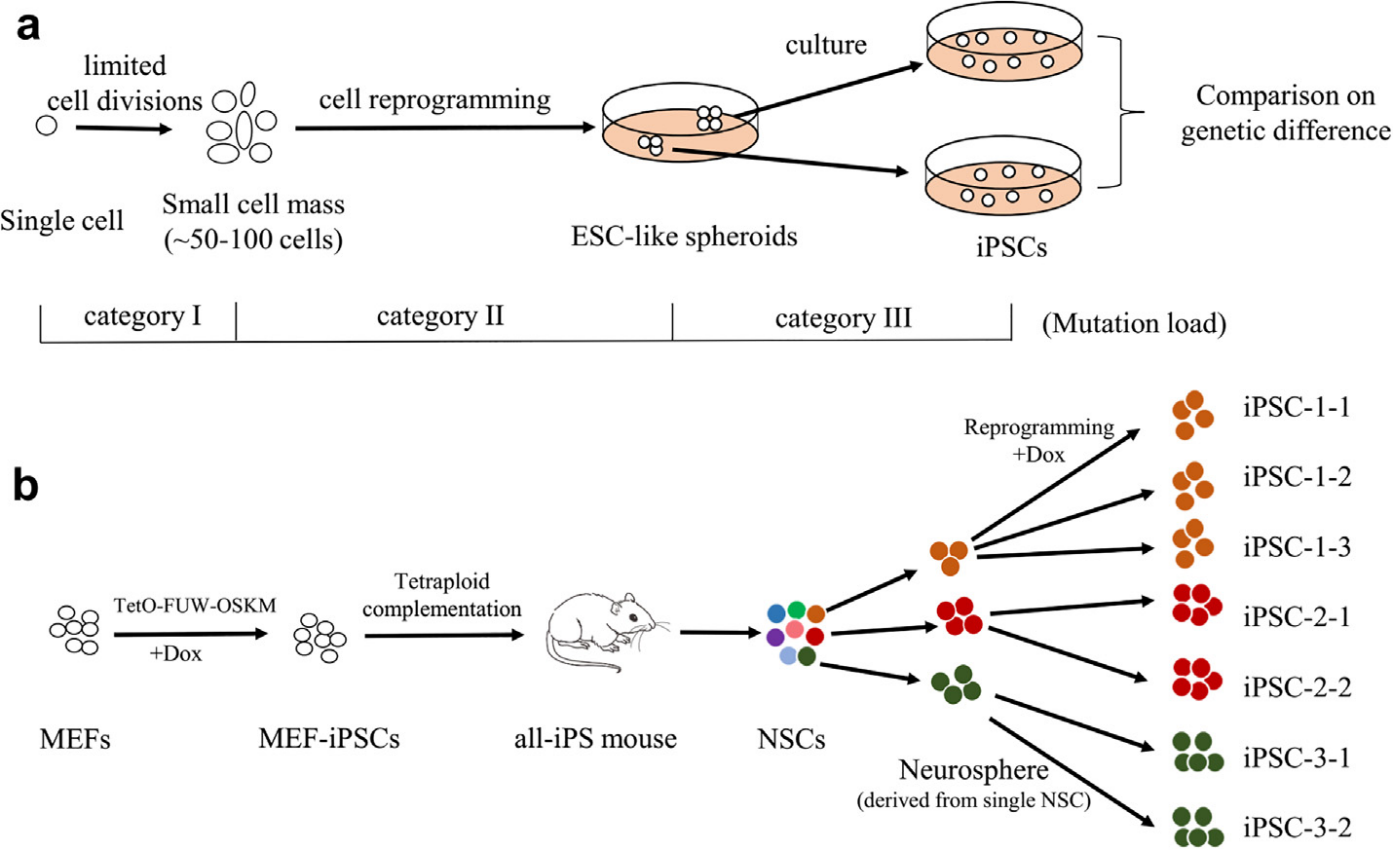
**A strategy and its experimental implementation to distinguish the composition of mutations observable in iPSCs. (a)** A design scheme to estimate the upper limit of the *de novo* mutations accumulated during reprogramming by limiting the pre-existing mutations from *in vivo* cell divisions to a small number. Category I, pre-existing mutations, those accumulated from *in vivo* cell divisions; category II, *de novo* mutations owing to iPS induction; and category III, mutations emergent from *ex vivo* iPSC culture expansion. **(b)** The experimental implementation and collection of the iPSC samples: iPSC-1 lines (iPSC-1-1, iPSC-1-2, and iPSC-1-3), iPSC-2 lines (iPSC-2-1 and iPSC-2-2), and iPSC-3 lines (iPSC-3-1 and iPSC-3-2). The MEF- iPSCs was established from mouse fibroblasts by retroviral introduction of TetO-FUW-Oct4, Sox2, Klf4 and c-Myc (OSKM). The all-iPS mouse was generated from the MEF-iPSCs through tetraploid complementation. Three individual single cells were isolated from neural stem cells (NSCs) of the newborn all-iPS mouse and formed three tiny neurospheres with 50-100 cells. Under Dox induction, the iPSC lines of (iPSC-1-1, iPSC-1-2, and iPSC-1-3), (iPSC-2-1 and iPSC-2-2), and (iPSC-3-1 and iPSC-3-2) were induced from the three neurospheres, respectively.

IPS induction, cell culture expansion, and cell division during tissue development *in vivo* all represent the sources of somatic variation in genomics. For simplicity, the observable mutations in iPSCs can be defined into three categories according to their origins: category I, pre-existing mutations, accumulated during *in vivo* cell divisions; category II, *de novo* mutations owing to iPS induction; and category III, mutations emergent from *ex vivo* culture expansion. To distinguish the compositions of these mutation categories in the iPSCs, we proposed a design scheme to estimate the upper limit of the mutations in the last two categories by limiting the mutations of category I to a small number (Fig. 1**a**). Specifically, a single somatic cell was isolated to grow into a tiny mass of cells during a very limited quantity of cell divisions, together with the accumulation of few somatic mutations for each cell in the cell mass. Then, no less than two iPSC lines were induced from each cell mass. Finally, the genetic differences which were composed chiefly of mutations in category II and III, with few category I mutations, were assessed between the pair of iPSC lines (Fig. 1**a**).

**Fig. 2:**
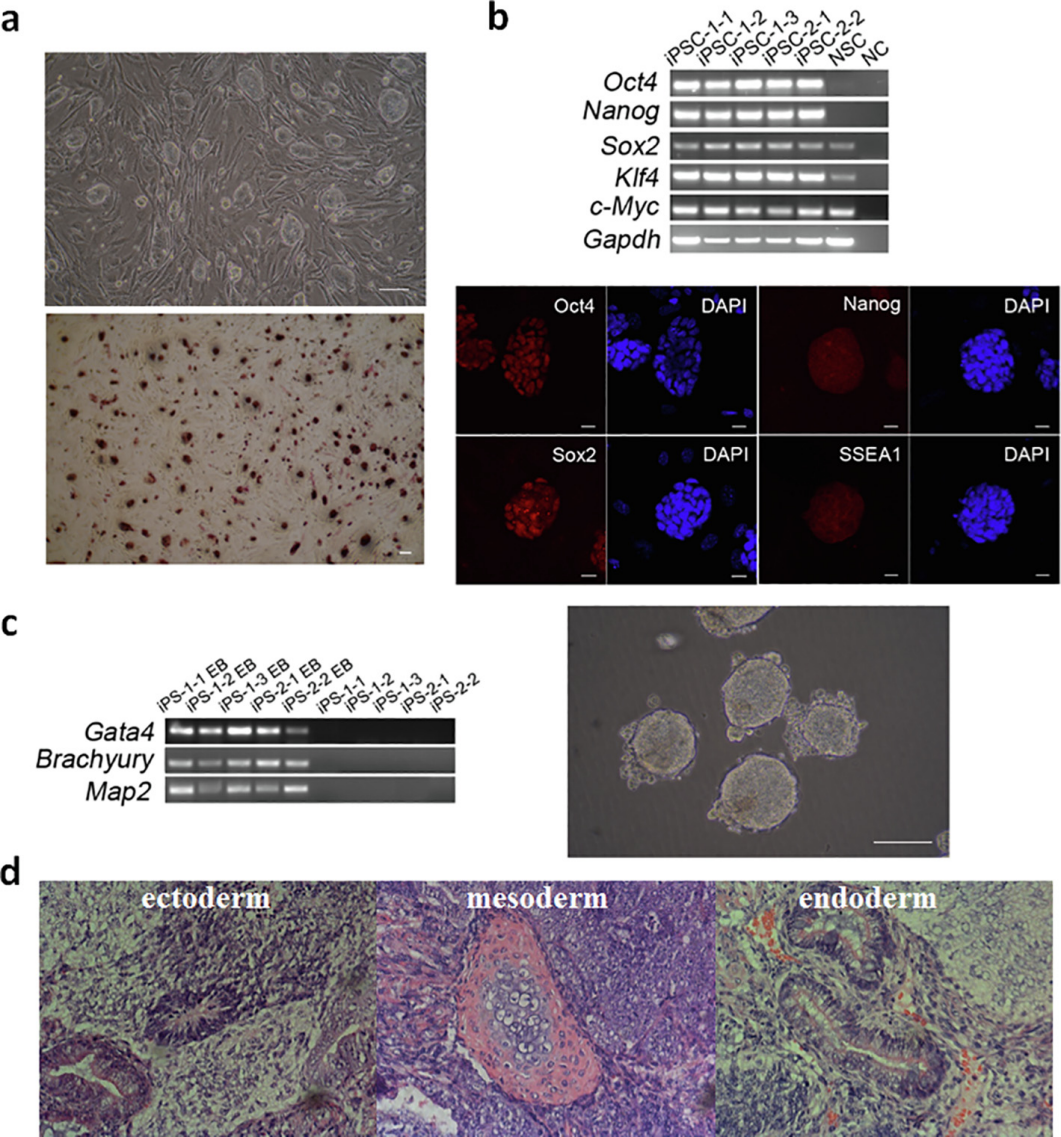
**Pluripotency characterization of iPSCs. (a)** Phase-contrast image and AP staining of the iPSC-1-2 cells. Scale bar, 100 μm. **(b)** RT-PCR of pluripotent markers for all the iPSCs. NSCs and template-free PCR systems (negative control, NC) were used as controls. And fluorescence immunostaining of pluripotency markers Oct4, Nanog, Sox2, and the ESC-specific surface marker SSEA-1 (red) for the iPSC-1-2 cells. Nuclei are stained with DAPI (blue). Scale bar, 20 μm. **(c)** RT-PCR of markers for 3-germ layers on day-6 embryoid bodies and embryoid body formation image for the iPSC-1-2 cells. Scale bar, 100 μm. **(d)** Teratoma formation of the iPSC-1-2 cells. The three-germ layers were detected by hematoxylin and eosin staining in a 3-week teratoma. (For interpretation of the references to colour in this figure legend, the reader is referred to the web version of this article.)

Experimentally, the all-iPS mouse was generated from the inducible MEF-iPSCs through tetraploid complementation, which expressed the endogenous tetracycline (Tet)-regulated four Yamanaka factors Oct4, Sox2, Klf4 and c-Myc [33]. Under doxycycline induction, iPSCs derived from any cells of the all-iPS mouse could be subsequently established with a high reprogramming efficiency. We isolated neural stem cells (NSCs) from a newborn all-iPS mouse. Three individual neural stem cells formed three tiny colonies with 50 ~ 100 cells, termed neurospheres. These neurospheres were then reprogrammed under doxycycline induction. The selected ESC-like clones, being far physical distance from each other in the cell culture dish, were picked and cultured into subsequent iPSC-1 lines (iPSC-1-1, iPSC-1-2, and iPSC-1-3), iPSC-2 lines (iPSC-2-1 and iPSC-2-2), and iPSC-3 lines (iPSC-3-1 and iPSC-3-2) **(**Fig. 1**b)**. At ten days after the addition of doxycycline to neurospheres, typical ESC-like morphological colonies emerged and were positive for alkaline phosphatase (AP) staining after propagation **(**Fig. 2**a,** Figure S1 **a)**. Pluripotency markers, including Oct4, Nanog, Sox2, SSEA-1, and others, were positively expressed in the iPSC lines as shown in RT-PCR and immunocytochemical staining images **(**Fig. 2**b,** Figure S1 **b)**. An embryoid body (EB) was successfully formed for each cell line that exhibited differentiation potential of three germ layers with the expressed markers of Gata4, Brachyury, and Map2 **(**Fig. 2**c,** Figure S1 **c)**. We further observed the formation of teratomas with three germ layers *in vivo* at three weeks after injection of iPSCs into severe combined immunodeficient (SCID) mice **(**Fig. 2**d,** Figure S1 **d)**. In addition, we detected the gene expression of another 9 markers for 3-germ layers, most of which were related to the later differentiation stage (Figure S1 e). Mesoderm marker genes, Eomes is involved in late-stage of gastrulation and differentiation of CD8 + T-cells, and Gata6 plays an important role in heart development. Ectoderm marker genes, Fgf5 is associated with hair elongation, and Pax6 and Nestin play important roles in the development of neural tissues and eye. Endoderm marker genes, FoxA1 is involved in the development of organ systems such as liver, pancreas, lung and prostate, Sox7 is associated with hemogenic endothelium differentiation, and Sox17 is required for gut endoderm development [34], [35]. As evidenced by the above cellular and molecular assay results, the induced cell lines derived from neurospheres maintained the stem-cell characteristics of proliferation and pluripotency.

### 3.2 Genomic comparisons of the paired iPSC lines profiling the composition of mutations

We applied whole-genome and exome sequencing to investigate the genomic profiles of the generated iPSC lines. The paired iPSC lines (iPSC-1-2, iPSC-1-3) at passage 7 and (iPSC-3-1, iPSC-3-2) at passage 4 achieved forty-fold coverage of whole-genome sequencing (WGS) data for each. Averaged forty-fold coverage of exome data was obtained for each iPSC line of the paired (iPSC-2-1, iPSC-2-2) at passage 7 and (iPSC-1-1, iPSC-1-3) at passage 7. We additional cultured and sequenced the iPSC-1-3 at passaged 16 to compare the genotype change of iPSCs during the multiple passaging procedures (Table 1). WGS data (forty-fold coverage) of the ancestral NSCs provided the most comprehensive collection of the germline background.

**Table 1:** Summary of the sequencing data and the genomic comparison results of the paired iPSC lines.

iPSCs	iPSC-1-1 (p7)^a^	iPSC-1-3(p7)^a^		iPSC-1-2 (p7)^a^	iPSC-1-3 (p16)^a^	iPSC-2-1 (p7)^b^	iPSC-2-2 (p7)^b^	iPSC-3-1 (p4)^c^	iPSC-3-2 (p4)^c^
Sequencing method	wes^d^	wes	wgs^d^	wgs	wgs	wes	wes	wgs	wgs
Genome coverage	60×	39×	41×	40×	45×	35×	39×	40×	37×
									
Observable SNVs in each iPSC line	15^e^(12)^f^	14(11)	1290 (1050)	1277 (1040)	1100 (895 )	9(7)	10(8)	1187 (966 )	1116 (908 )
									
	iPSC-1-1(p7) vs. iPSC-1-3(p7)		iPSC-1-3(p7) vs. iPSC-1-2(p7)		iPSC-1-3 (p16) vs.(p7)	iPSC-2-1(p7) vs. iPSC-2-2(p7)		iPSC-3-1(p4) vs. iPSC-3-2(p4)	
					
Unique SNVs in each of paired iPSC lines	1^e^(1)^f^	0(0)	55(45)	42(34)	21(17)	1(1)	2(2)	160(130 )	89(72)

^a^iPSCs derived from single-cell #1(p7: passage 7, p16: passage 16).

^b^iPSCs derived from single-cell #2.

^c^iPSCs derived from single-cell #3.

^d^wes: whole exome sequencing, wgs: whole genome sequencing.

^e^Called SNVs with NSCs as a control.

^f^Corrected SNV numbers by Sequenom validation.

The subsequent analysis of WGS data elucidated the full profiles of SNVs observable in the paired iPSC lines. After SNV calling, we observed 42 SNVs uniquely present in iPSC-1-2 and 55 in iPSC-1-3 (Table 1**, Table S1**). The two iPSC lines shared 1235 SNVs when we utilized the parental NSCs as a source of germ-line control (Table 1**, Table S1**). Together with all the observed SNVs in coding regions, randomly sampled non-coding SNV loci were validated via Sequenom genotyping (**Table S2**). The genotyping results defined the relevant true positive rates (81.4%) of SNV calling, which were used to correct the unique SNV numbers of iPSC-1-2 and iPSC-1-3 to be 34 and 45 ones, respectively (Table 1). Similarly, the SNV number shared by the paired iPSC lines (iPSC-1-2, iPSC-1-3) but specific to parental NSCs was corrected to be 1005 (Table 1). We thus concluded that the *de novo* SNVs in category II and III across the whole genome were<34 and 45; i.e., < 3.3% (34/1040) and 4.3% (45/1050), respectively, of the observable SNVs in iPSC-1-2 and iPSC-1-3. The genomic comparison result between paired iPSC lines (iPSC-3-1 and iPSC-3-2) was also summarized in the table 1. This paired iPSC lines exhibited dozens of specific SNVs, whereas there were about one thousand shared SNVs. Comparison and validation on exome data of another two pairs of iPSC lines, (iPSC-2-1, iPSC-2-2) and (iPSC-1-1, iPSC-1-3) were additionally performed. In the exome, the paired iPSC lines exhibited less than two SNVs that were distinct from each other, whereas there were tens of common SNVs shared by the paired iPSC lines (Table 1, **Table S1**, **Table S2**). The above evidence supported the conclusion that *de novo* SNVs accumulated during reprogramming accounted for a small proportion of the total observable SNVs in iPSCs. While the two iPSC lines of iPSC-1-3 at passage 7 and at passage 16 shared 1079 SNVs as compared with parental NSCs, there were 21 SNVs uniquely present at the passage 16 (Table 1**)**. No specific copy number aberrations occurred in the iPSCs at passage 7 and passage 16 (Figure S2). The result demonstrated that the genotypes of the iPSC-1-3 were almost identical during the multiple passaging procedures.

**Fig. 3:**
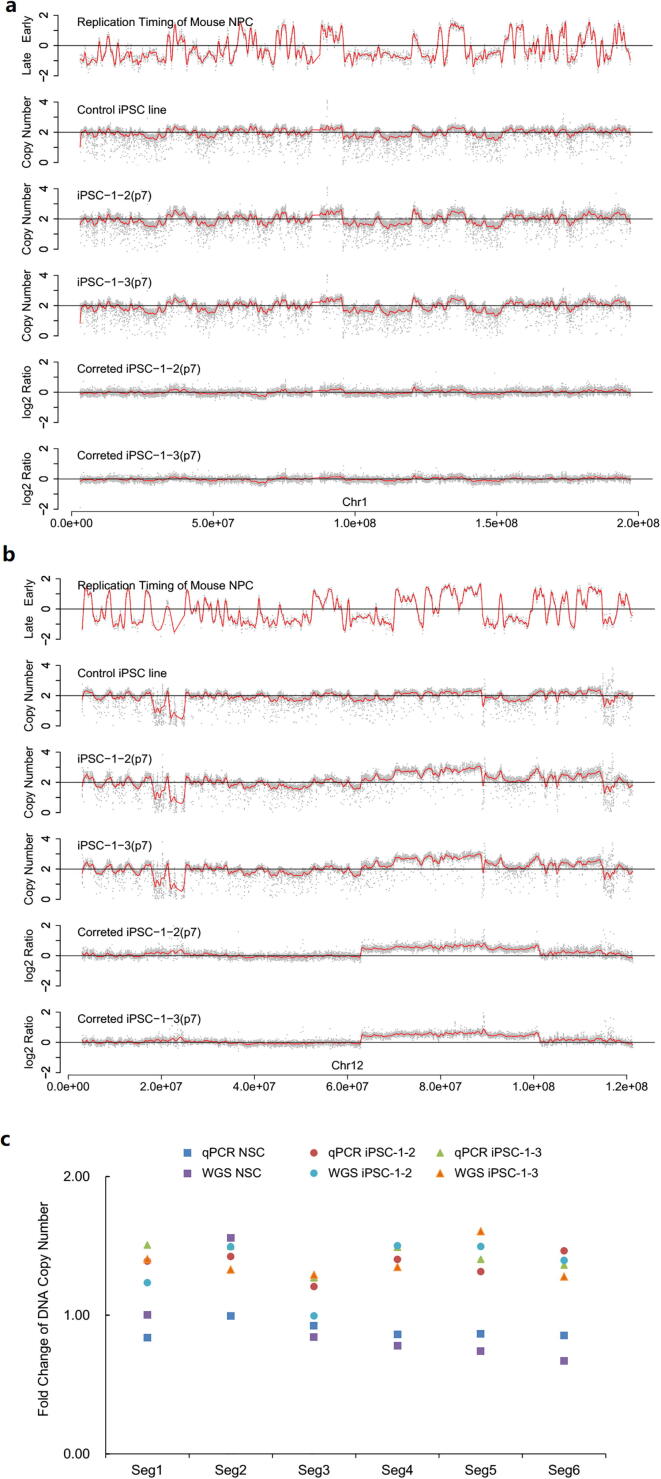
**Copy number profiles in the paired iPSC lines (iPSC-1**-**2 and iPSC-1**-**3). (a)** Chromosomal 1: The replication timing profile (log2(Early/Late)) of mouse neural progenitor cell (NPC); the read depth profiles of the paired iPSC lines (iPSC-1-2 and iPSC-1-3) at passage 7 and the control iPSC line, with consistent pseudo copy number aberrations (CNAs) owing to replication timing; and the corrected log2-ratio copy number profiles of the paired iPSC lines after excluding the pseudo effect of replication timing. **(b)** Chromosome 12. There is one gain (~38 M length) shared by the paired iPSC lines (iPSC-1-2, iPSC-1-3) at passage 7. (**c**) The qPCR validation result for the copy number aberration on Chromosome 12. Six segments (Seg1-Seg6) on this CNA and four non-CNA regions were verified via qPCR. Fold changes of DNA copy number were estimated from qPCR data and WGS data in the samples of iPSC-1-2 (passage 7), iPSC-1-3 (passage 7), NSCs and the control iPSC line.

The sequencing data further revealed the genome-wide read-depth profiles of the iPSC lines. The timing of replication, which measures the temporal order of replication, introduces distinct DNA dosages during S phase of the cell cycle [36]. Accordingly, all the read-depth profiles exhibited the same “gain” or “loss” patterns as that of the replication timing profile, because of a significantly extended S phase in pluripotent cells (Fig. 3**, and** Figure S2) [33]. After normalization with the read-depth profile of an iPSC line, the pseudo “gain” or “loss” effect caused by replication timing was excluded in the corrected log2 ratio read-depth profiles of iPSCs (Fig. 3 **and** Figure S2). The mutations specific in one of the paired iPSC lines would arise during or after reprogramming. There was one gain on chromosome 12 (~38 M length) shared by the paired iPSC lines (iPSC-1-2, iPSC-1-3), but being not present in NSCs. It arose during *in vivo* cell divisions of all-iPS mouse NSC as a pre-existing somatic mutation, instead of the occurrence during or after reprogramming. The qPCR values for validation on this gain on chromosome 12 were consistent with the copy numbers estimated from WGS data (Fig. 3**c**). No apparent *de novo* copy number aberrations (CNAs) were observed (Fig. 3**a**, Fig. 3**b**, and Figure S2).

### 3.3 The clonal expansion representing the origin of each iPSC line from an individual somatic cell

**Fig. 4:**
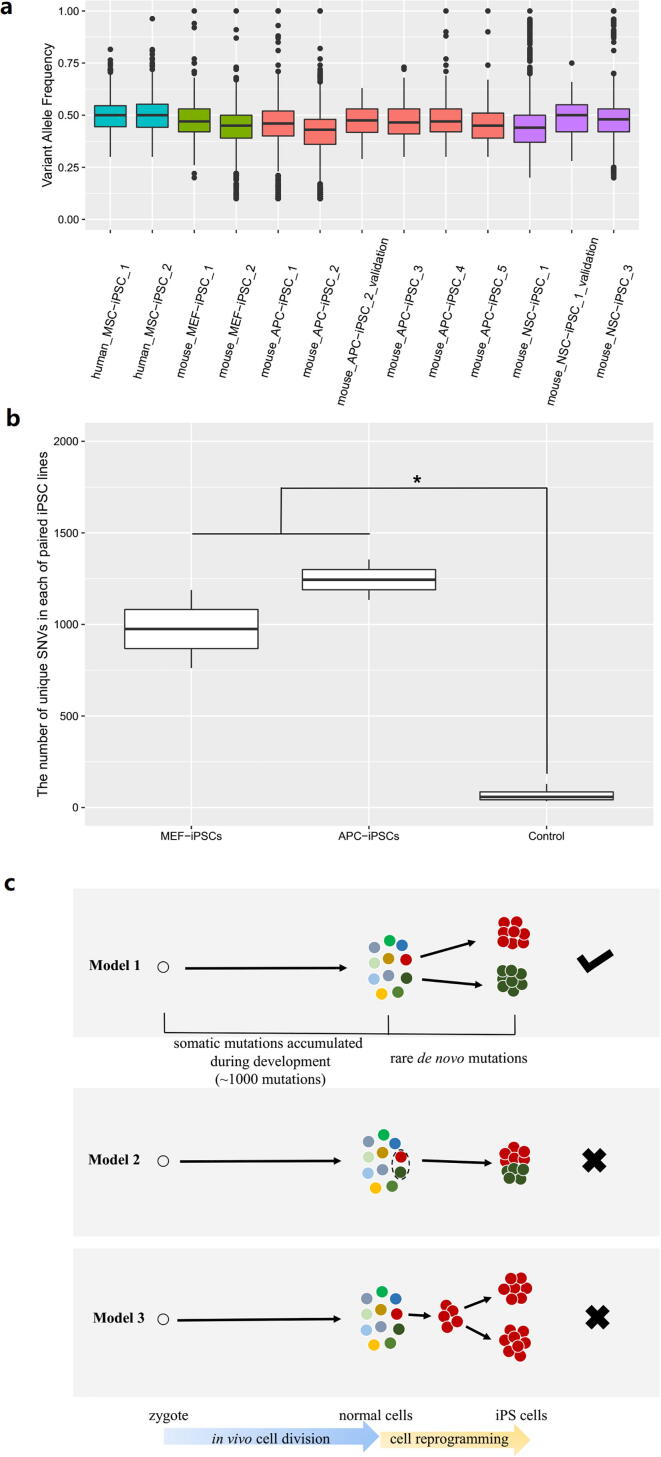
**Clonal expansion of each iPSC line from an individual somatic cell revealed by the mutation profiles. (a)** Variant allele frequency spectra (box plot) of SNVs observable in multiple iPSC lines. The iPSC lines were induced from cell types of human mesenchymal stem cell (MSC), muse neural stem cell (NSC), mouse embryonic fibroblast (MEF) and mouse adipocyte progenitor cell (APC), named as human_MSC_iPSC_1 and so on. **(b)** The numbers of unique SNVs in each of paired iPSC lines induced in one regular reprogramming experiment. MEF-iPSCs: paired iPSC lines induced from MEF in one regular reprogramming experiment; APC-iPSCs: paired iPSC lines induced from APC in another regular reprogramming experiment; Control: estimated upper-limit amount of *de novo* SNVs owing to iPS induction and subsequent culture expansion. *: significantly different in statistics ith P-value < 0.0001. **(c)** Three models describing the clonal expansion of cells and corresponding mutation accumulation during the entire process, beginning from the zygote, to somatic cells, to the resultant iPSCs. The model #1 is an accepted model and the model #2 and #3 are rejected.

To address whether each iPS cell line is clonally derived from a unique somatic cell, we analyzed the frequency spectra of the observable SNVs in iPSCs. A collection of box-plots showed variant allele frequency spectra of multiple iPSC lines induced by distinct reprogramming strategies or experiments (Fig. 4**a**, **Table S3**). The frequency spectra with a median value of the frequencies close to 0.5, indicating that the SNVs presented in nearly all cells of each iPSC line, defined the induction of clonal expansion from a single-cell. We further analyzed the allele frequency spectra of genomic integration of a four-factor vector in other iPSC lines induced by a four-factor-integration reprogramming strategy. The allele frequencies of vector integration sites were also near to 0.5, which revealed that the occurrence of the single-cell clonal expansion was not earlier than the beginning time point of cell reprogramming. On the basis of it, we rejected the model #2 that described a type of cell expansion pattern from somatic cells to the iPSCs (Fig. 4**c**). In summary, the mutation allele frequency of 0.5 represents the single-somatic-cell origin and the subsequent clonal expansion of each iPSC line. This inference is consistent with the routine experimental protocols of cell reprogramming wherein clonal ESC-like spheroids were manually picked and cultured to become an iPSC line. Thus, the pre-existing somatic mutations in the single somatic cell were then inherited as the genetic background of all the progeny cells.

Another question was subsequently raised regarding whether any two clonal ESC-like spheroids, which were picked from the same medium and cultured into two iPSC lines, were derived from two individual somatic cells during a regular reprogramming experiment. Using public data, we performed genomic investigation on several iPSC lines induced in the same regular reprogramming experiment (Fig. 4**b**). The above argument was proved to be true, as the result of SNV comparisons demonstrated that the SNVs specific in each iPSC line was much more than the upper-limit amount of *de novo* SNVs we estimated (Fig. 4**b**, **Table S3**). Therefore, the model #3 that described another cell expansion pattern from somatic cells to the iPSCs was rejected (Fig. 4**c**).

When we reviewed the entire process, beginning from the zygote, to somatic cells, to the resultant iPSCs, an accepted model (model #1) describing the dynamic cell lineage accompanied by the accumulation of mutations was developed (Fig. 4**c**). Hundreds of somatic SNVs accumulate per cell division from a zygote to a somatic cell but are unobservable because of the absence of clonal expansion. Induced cell reprogramming introduces few somatic mutations and enables the single somatic cell to become a unique iPSC clone. The model supports the conclusion that iPSC-based cell reprogramming is a sensitive and specific cell cloning strategy that amplifies the DNA of each single cell for the purpose of somatic mutation screening.

### 3.4 Characteristics of somatic SNVs in heterogeneous normal cells

Advances in single-cell DNA amplification technology have made it possible to collect somatic SNVs and analyze their characteristics in heterogeneous normal cells. In particular, current studies have released genome sequencing data for many iPSCs from various types of somatic cells, which have efficiently explored the somatic mutations of the corresponding single somatic cell from the genomic profile of each iPSC line. These somatic cell types include mesenchymal stem cell (MSC), neural stem cell (NSC), mouse embryonic fibroblast (MEF) and adipocyte progenitor cell (APC).

**Fig. 5:**
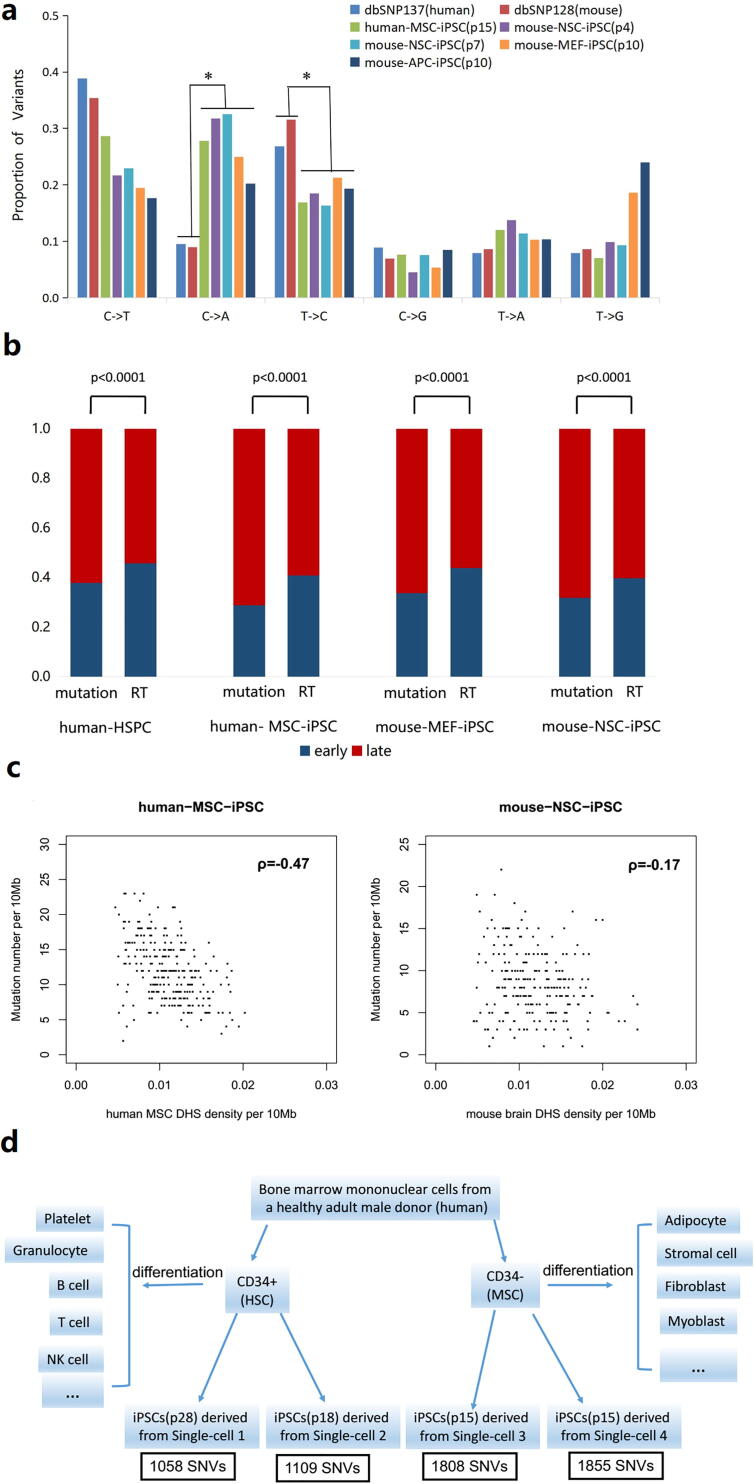
**Characteristics of somatic SNVs in heterogeneous normal cells. (a)** The somatic SNV spectra in normal cells (MSC, NSC, MEF and APC), comparing with the SNP spectra. The SNP information of Human and Mouse was retrieved from the dbSNP137 and dbSNP128, respectively. “*” denotes a significant difference in statistics. **(b)** The distribution of somatic SNVs in early and late replication timing regions. The control bar (named as RT) denotes the length ratio of early replication timing regions to late regions for a certain cell type. **(c)** Negative correlation of mutation density and DHS density calculated using the Spearman’s rank correlation coefficient (ρ). **(d)** Distinct mutation loads in two subtypes of bone marrow mononuclear cells, the CD34 + cells and the CD34- cells, that have different differentiation potentials.

We summarized the SNV spectra of four types of normal cells as well as the SNP spectra (Fig. 5**a**, **Table S3**). The SNV spectra of normal cells, unrelated to cell type and species, were in good agreement with each other but distinct from the SNP spectra. Besides a C-to-T predominance, the spectra of SNVs in normal cells also show a C-to-A predominance. In contrast to the C-to-A predominance, there is a low proportion of C-to-A in the spectra of germ-line nucleotide substitutions (Fig. 5**a**).

Furthermore, the quantity of somatic SNVs was found to be statistically associated with replication timing in various cell types. In addition to the SNVs observed in stem-cell clonal HSPCs (Hematopoietic stem/progenitor cells), the somatic SNVs observed in MSCs, MEFs, and NSC-derived iPSCs significantly occurred in late replicating regions (Fig. 5**b**). Moreover, the density of DNase I hypersensitive sites (DHS) and the occurrences of somatic SNVs in MSCs and NSCs were negatively correlated based on a Spearman correlation coefficient calculation (Fig. 5**c**), consistent with the previous study [6]. This pattern implied the association between the somatic mutation density and the chromatin distribution.

The CD34 antibody marks the bone marrow mononuclear cells into two subtypes of the CD34 + cells, consisting primarily of Hematopoietic stem cells (HSCs), and the adherent CD34- cells, composed chiefly of MSCs. We surveyed the load of somatic SNVs in both single CD34 + cells and single CD34- cells based on the whole-genome sequencing data of the CD34 + cell-derived and the CD34- cell-derived iPSCs from an adult healthy male. The SNV load (~1800) in each CD34- cell was significantly 1.8 fold of that (~1000) in each CD34 + cell (Fig. 5**d**, **Table S3**). The data suggest that two subtypes of adult stem cells with different differentiation potential could have distinct mutation loads, even though they were collected from the same tissue resource.

## 4 Discussion

Comparing with the parental cells, researchers always observe many somatic mutations in pluripotent cells reprogrammed by iPS technique. However, it is still unclear how many mutations are induced *de novo* during reprogramming in all the observable mutations. We have designed an experiment to help estimate the upper limit of the *de novo* mutations owing to iPS induction and subsequent culture. It is notable that the procedure of limiting the pre-existing mutations from *in vivo* cell divisions to a small number is of importance. A previous comparable experiment using endothelial progenitor cells (EPCs) had a different conclusion due to excessive pre-existing mutation accumulation in the cell mass propagated through multiple cell divisions from a single cell [37]. In our experiment, a doxycycline-regulated lentiviral vector was inserted into the cell genomes and then Tet-on/off control of TetO-FUW-Oct4, Sox2, Klf4, and c-Myc triggered the reprogramming process [33]. Our study demonstrates that most of the observable mutations in iPSCs are pre-existing and meanwhile few mutations occur during reprogramming.

In this study, we provided evidence to prove that iPSCs, being a clonal cell population of an individual somatic cell, records almost identical mutational profile of its initial cell progenitor. Therefore, the traditional iPS technique meets a new and effective design for single-cell genomic analysis by means of cell-clonal amplification of the single cell’s DNA copy. In detailed protocol, somatic cells are reprogrammed into cells with the characteristic of proliferation after induction with or free of genomic integration of exogenous reprogramming factors; Secondly, ES-like colonies from distinct single cells appear in the medium and are then individually cultured till full-dose DNAs are clonal duplicated; Lastly, bulk DNA sequencing and subsequent mutation analysis reveals the full genomic profile of each initial single cell.

Single-cell genomic analysis has advanced rapidly with the two most common amplification strategies being single stem cell-derived clonal culture or organoid formation and pg-level DNA amplification using reaction reagents [11], [38]. Currently, the former is generally accepted as a “golden” strategy to evaluate the mutational profile of a single cell because of high-fidelity DNA amplification. The technology relative to reaction-reagent based pg-level DNA amplification has been improved to reduce DNA contamination, uneven genomic coverage and allele dropout. But even for the latest method LIANTI and SCMDA, hundreds of false-positive and false-negative SNVs could been introduced when it was used to survey the genome-wide SNV profile of a single cell [11], [12]. By comparison, our iPS-based cell cloning strategy has almost equivalent performance as the golden strategy except for introducing dozens of SNVs genome-wide as the false-positives. Moreover, our strategy can be applicable to any cell type, not merely to stem cells, with significantly shortened experimental period of 3 weeks (1/3-1/2 time of the organoid formation technology [38]. Both the acceptable reprogramming efficiency and the “stochastic” feature of direct cell reprogramming, which make each somatic cell have an equal chance to be efficiently reprogrammed into iPS cells, further promise the feasibility of widespread use of the iPS-based single-cell cloning strategy [39], [40]. The suitability of iPSCs for subsequent genome engineering and directional differentiation could promotes the functional studies on the biological effects of somatic mutations in single cells.

The origin of somatic mutations in human iPSCs is important with regard to therapeutic delivery of differentiated cells derived from human stem or iPS cells. In current work, we studied the origin of somatic mutations in mouse iPSCs based on a designed experimental system that could derive paired iPSCs from two individual parental cells in a tiny cell colony. Thus the observable mutations in such iPSCs were composed of *de novo* mutations during reprogramming and cell culture, and few pre-existing mutations. The upper limit of *de novo* mutations in iPSCs could be estimated by comparison on genetic differences between the paired iPSCs. We believe the results about the origin of somatic mutations in mouse iPSC progeny, to some extent, would guide the understanding of mutation composition in human iPSCs. Unfortunately, it is difficult to design the same experimental system for similar research on human iPSCs. The first reason is the difference of reprogramming efficiency between human and mouse cells via exogenous transduction of Yamanaka factors. The reprogramming efficiency of mouse cell is 0.1%–1% [41], while that of human cell is relatively low (0.01%) [42], [43]. In our experiment, we additionally increased reprogramming efficiency with the utility of the parental cells from all-iPS mouse that expressed endogenous Yamanaka factors so that we could successfully induce two or more iPSCs from 50 ~ 100 cells in a tiny cell colony. Secondly, a single somatic cell that could *ex vivo* grow into a tiny cell mass is required. Such a single somatic cell can be obtained by dissection and isolation of some adult stem cells from fetal mice. For example, we isolated a neural stem cell to form a tiny neurosphere as the source for reprogramming. But the lack of clinical environment made it a pity not to obtain adult stem cells from human samples. In addition, human or mouse iPSCs are typically derived from differentiated “older” somatic cells than neural stem cells. Whether the “older” parental cells might be prone to mutagenesis is a matter of concern.

## CRediT authorship contribution statement

**Xuexia Miao:** Methodology, Software, Formal analysis, Investigation, Writing - original draft. **Yueying Li:** Methodology, Validation, Investigation, Writing - original draft. **Caihong Zheng:** Investigation, Data curation, Funding acquisition. **Lifei Wang:** Software. **Chen Jin:** Resources. **Lei Chen:** Validation. **Shuangli Mi:** Validation. **Weiwei Zhai:** Data curation. **Qianfei Wang:** Conceptualization, Methodology, Supervision, Writing - original draft, Funding acquisition. **Jun Cai:** Conceptualization, Methodology, Supervision, Writing - original draft, Funding acquisition.

## Declaration of Competing Interest

The authors declare that they have no known competing financial interests or personal relationships that could have appeared to influence the work reported in this paper.
