# Supplementary material for: A promising iPS-based single-cell cloning strategy revealing signatures of somatic mutations in heterogeneous normal cells
Source: Comput Struct Biotechnol J. 2020 Sep 3;18:2326–35. doi: 10.1016/j.csbj.2020.08.026 (PMC7493045; doi:10.1016/j.csbj.2020.08.026)
Supplement: Supplementary data 1 [file mmc1.pdf]

Figure S1. Characterization of four iPSCs cell clones (passage 7): iPSC-1-1, iPSC-1-3, iPSC-2-1 and iPSC-2-2. Related to Figure 2. (a) Phase-contrast image and AP staining. Scale bar, 100 μm. (b) Fluorescence immunostaining of pluripotency markers Oct4, Nanog, Sox2, and the ESC-653 specific surface marker SSEA-1 (red). Nuclei are stained with DAPI (blue). Scale bar, 20 μm. (c) Embryoid body formation image. Scale bar, 100 μm. (d) Teratoma formation. Cell types of the three germ layers were detected by hematoxylin and eosin staining in a 3-week teratomas. (e) qRT-PCR showed the differentiation marker gene expression of the three germ layers on day-6 embryoid bodies. Mesoderm marker genes: Brachury, Eomes, Gata6; Ectoderm marker genes: Fgf5, Pax6, Nestin; Endoderm marker genes: Sox7, Sox17, FoxA1. Data are shown as means±SEM, n=3. $*p < 0.05$, $**p < 0.01$.

Figure S2. Genome-wide replication timing and copy number pattern of all the chromosomes in all iPSCs. Related to Figure 3.

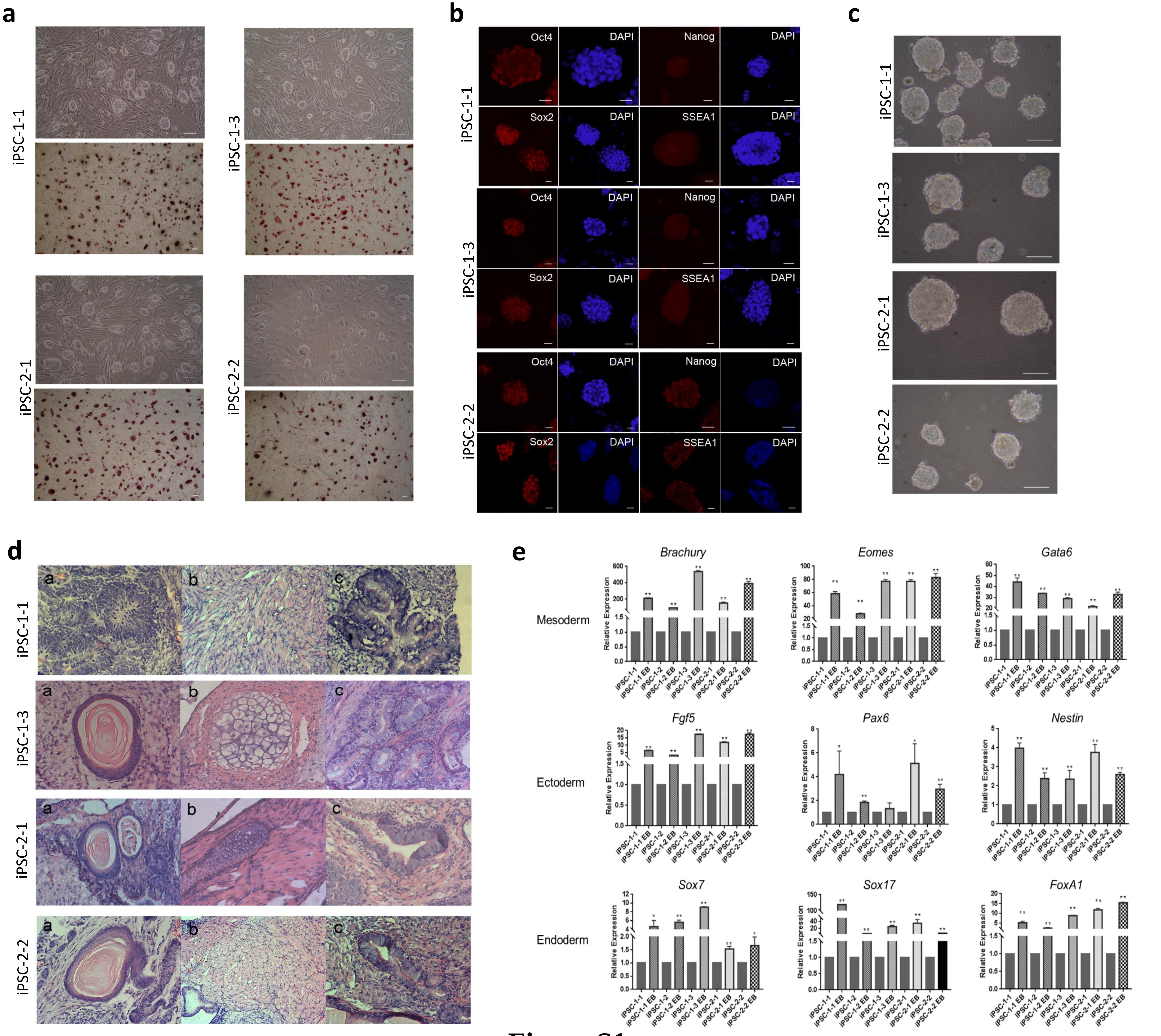
a
iPSC-1-1
iPSC-1-3
iPSC-2-1
iPSC-2-2
b
Oct4
DAPI
Nanog
Sox2
SSEA1
c
d
e
Brachury
Eomes
Gata6
Fgf5
Pax6
Nestin
Sox7
Sox17
FoxA1
Mesoderm
Ectoderm
Endoderm
Relative Expression

Figure S1

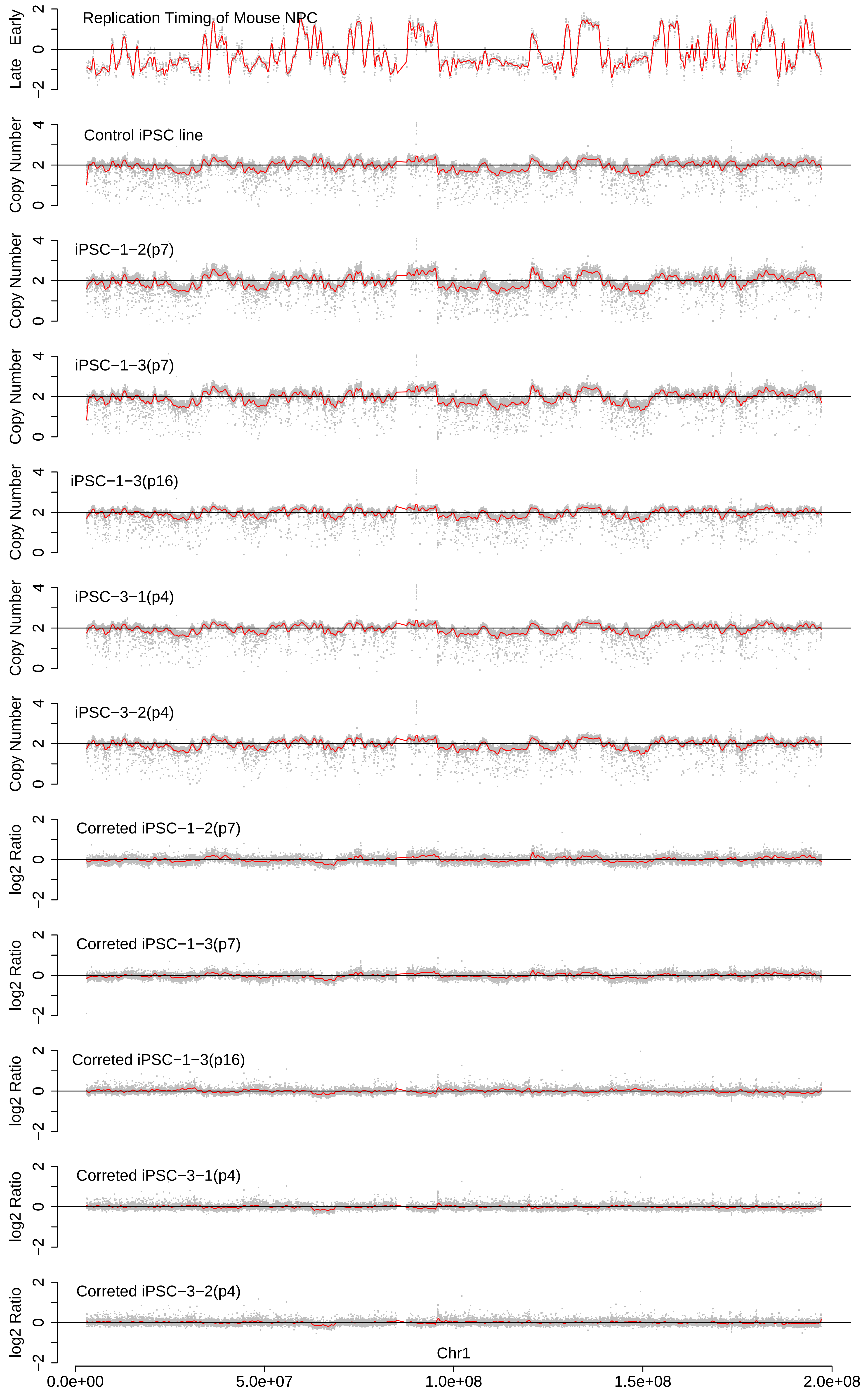
Replication Timing of Mouse NPC
Early
Late
Control iPSC line
iPSC−1−2(p7)
iPSC−1−3(p7)
iPSC−1−3(p16)
iPSC−3−1(p4)
iPSC−3−2(p4)
Correted iPSC−1−2(p7)
Correted iPSC−1−3(p7)
Correted iPSC−1−3(p16)
Correted iPSC−3−1(p4)
Correted iPSC−3−2(p4)
Copy Number
log2 Ratio
Chr1
0.0e+00
5.0e+07
1.0e+08
1.5e+08
2.0e+08

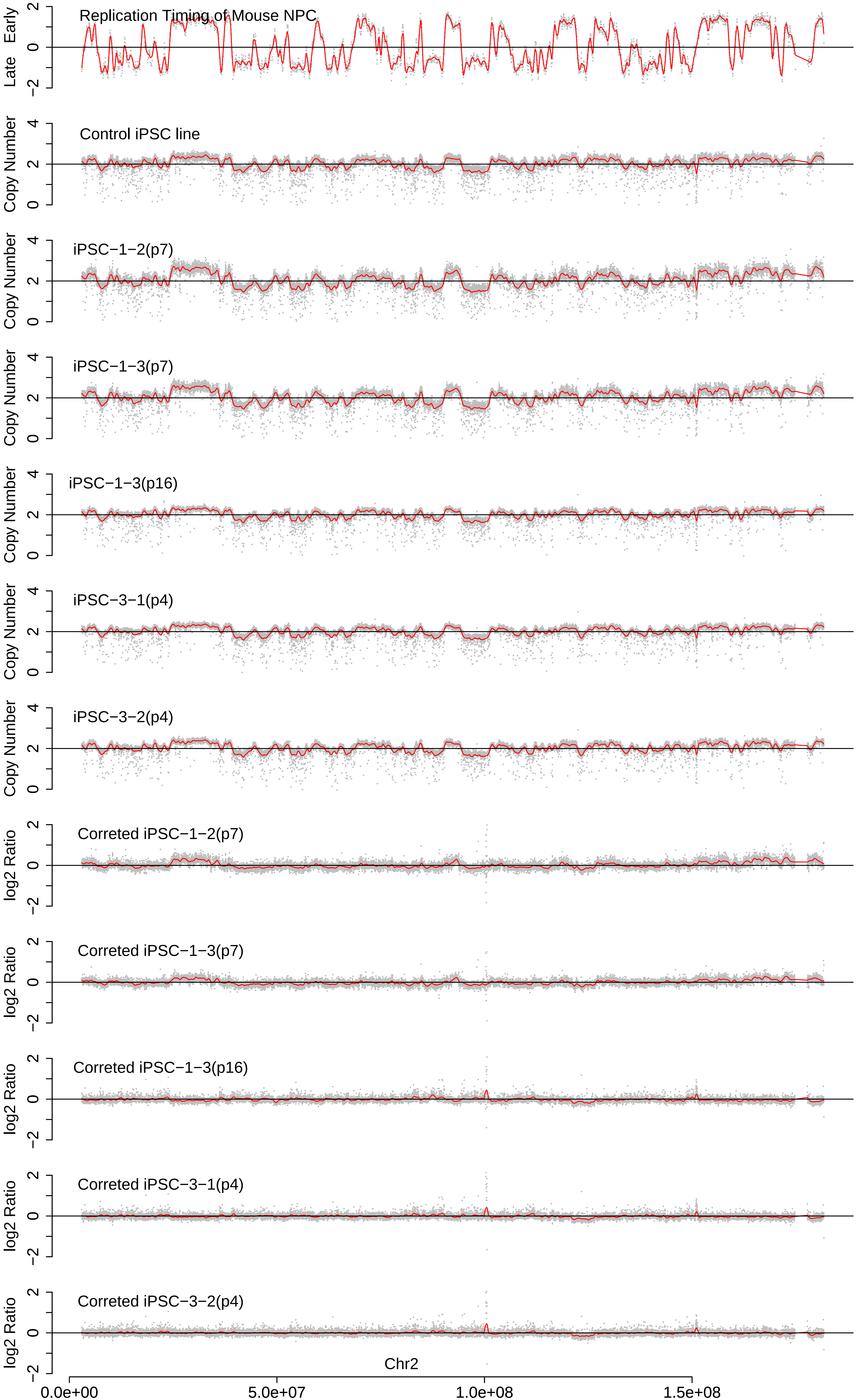

Replication Timing of Mouse NPC
Early
Late
Control iPSC line
iPSC−1−2(p7)
iPSC−1−3(p7)
iPSC−1−3(p16)
iPSC−3−1(p4)
iPSC−3−2(p4)
Copy Number
Correted iPSC−1−2(p7)
Correted iPSC−1−3(p7)
Correted iPSC−1−3(p16)
Correted iPSC−3−1(p4)
Correted iPSC−3−2(p4)
log2 Ratio
Chr2
0.0e+00
5.0e+07
1.0e+08
1.5e+08

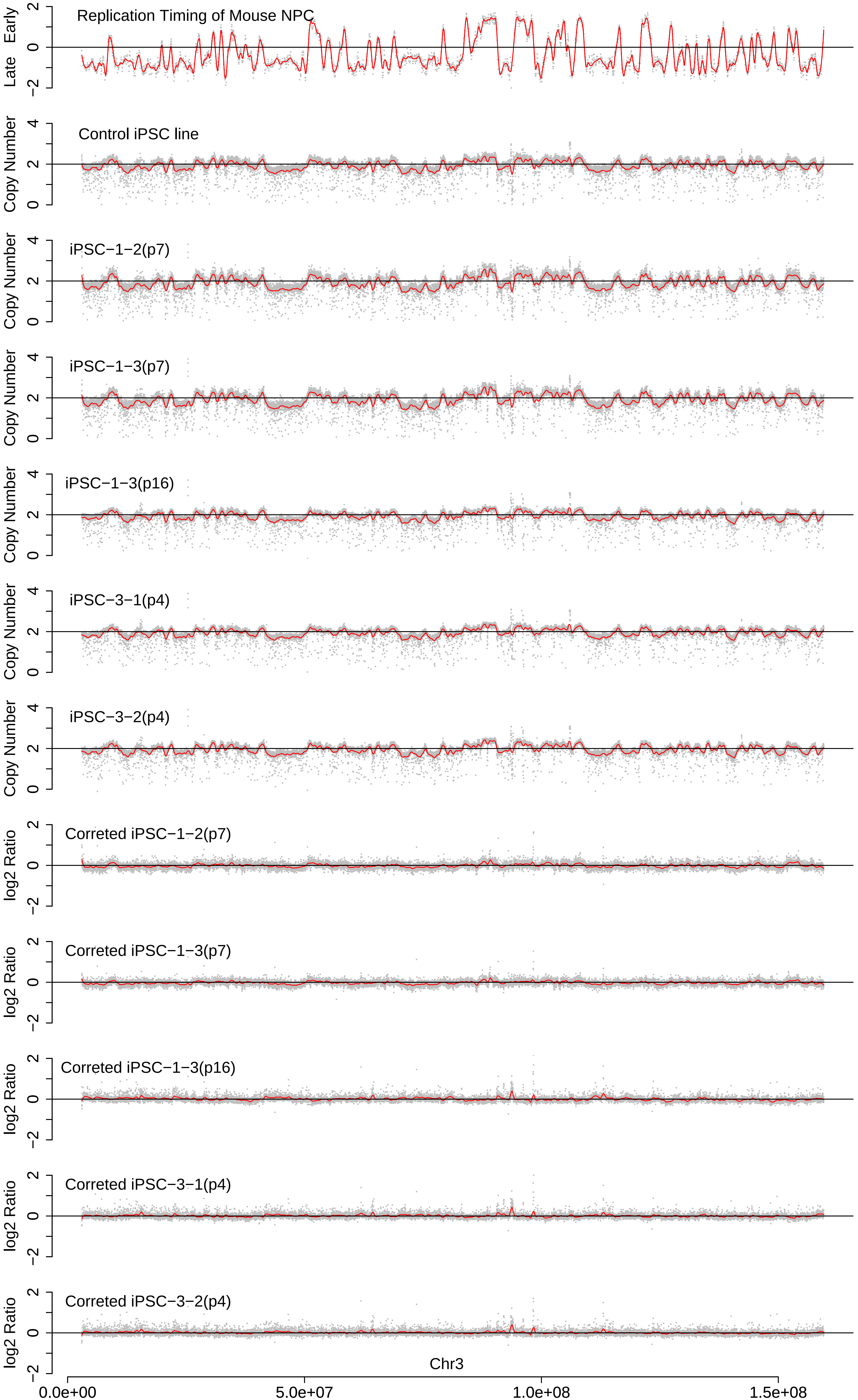
Replication Timing of Mouse NPC
Early
Late
Control iPSC line
iPSC−1−2(p7)
iPSC−1−3(p7)
iPSC−1−3(p16)
iPSC−3−1(p4)
iPSC−3−2(p4)
Corrected iPSC−1−2(p7)
Corrected iPSC−1−3(p7)
Corrected iPSC−1−3(p16)
Corrected iPSC−3−1(p4)
Corrected iPSC−3−2(p4)
Copy Number
log2 Ratio
Chr3
0.0e+00
5.0e+07
1.0e+08
1.5e+08

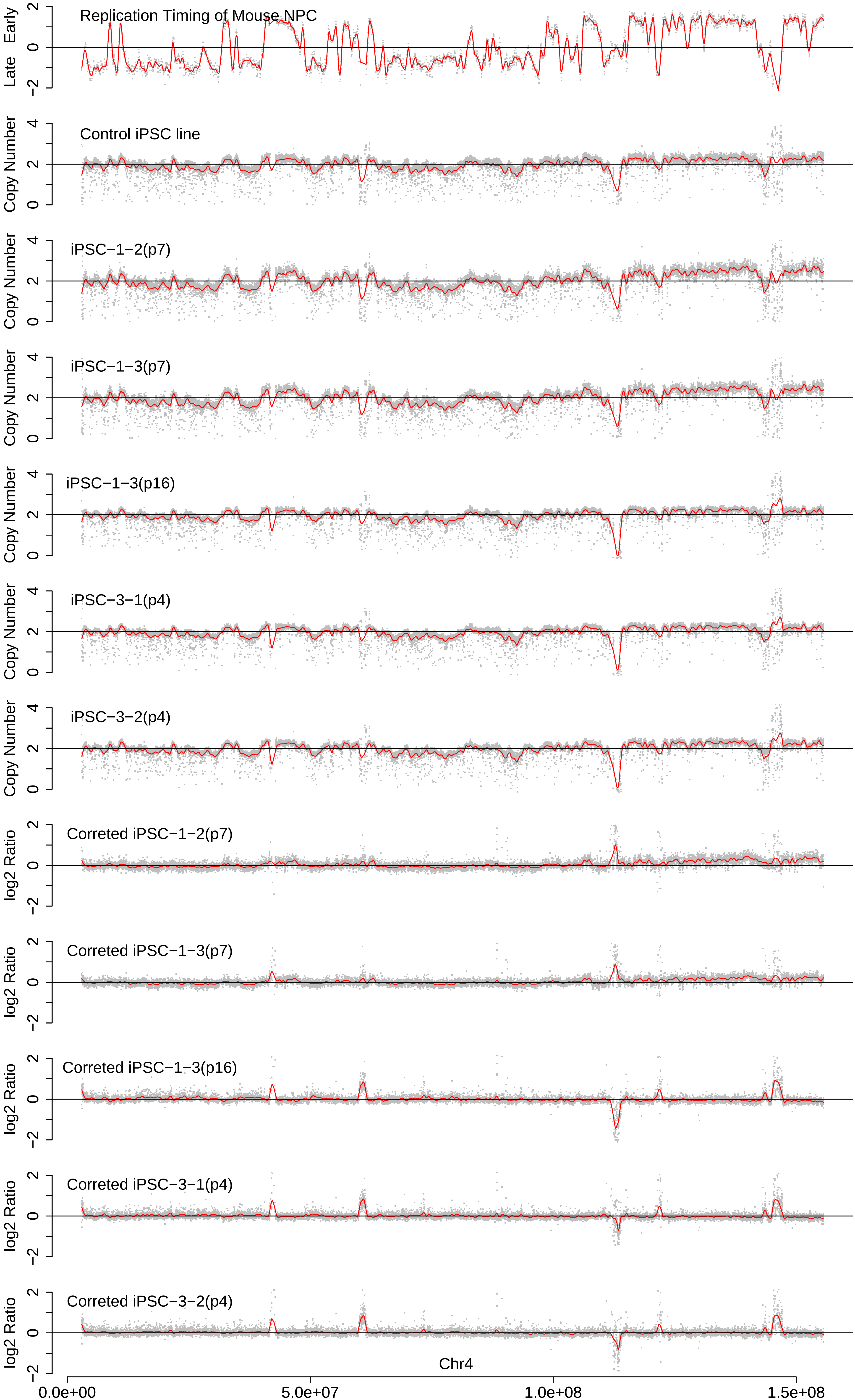

Replication Timing of Mouse NPC
Early
Late
Control iPSC line
iPSC−1−2(p7)
iPSC−1−3(p7)
iPSC−1−3(p16)
iPSC−3−1(p4)
iPSC−3−2(p4)
Copy Number
Correted iPSC−1−2(p7)
Correted iPSC−1−3(p7)
Correted iPSC−1−3(p16)
Correted iPSC−3−1(p4)
Correted iPSC−3−2(p4)
log2 Ratio
Chr4
0.0e+00
5.0e+07
1.0e+08
1.5e+08

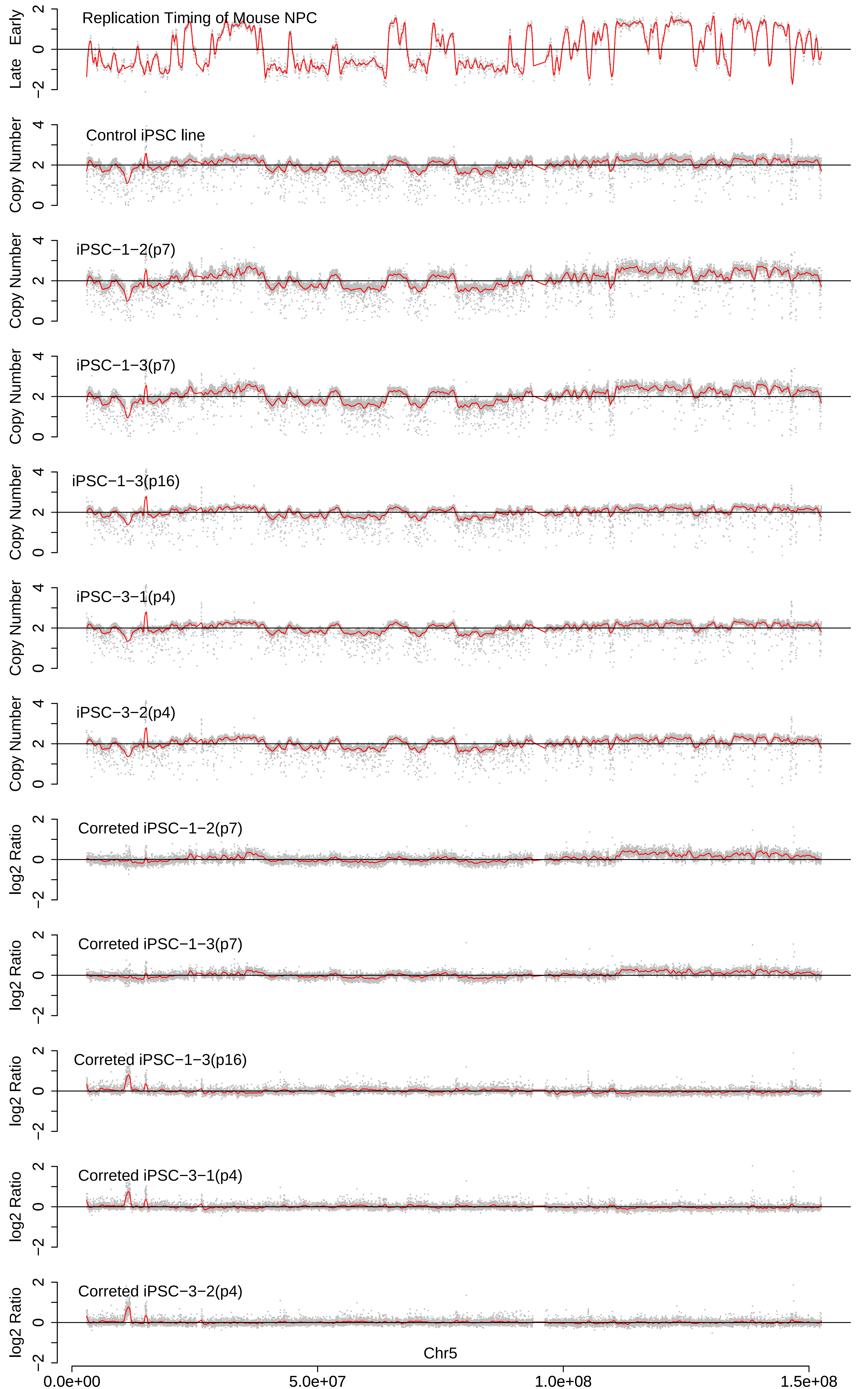

Replication Timing of Mouse NPC
Early
Late
Control iPSC line
iPSC−1−2(p7)
iPSC−1−3(p7)
iPSC−1−3(p16)
iPSC−3−1(p4)
iPSC−3−2(p4)
Corrected iPSC−1−2(p7)
Corrected iPSC−1−3(p7)
Corrected iPSC−1−3(p16)
Corrected iPSC−3−1(p4)
Corrected iPSC−3−2(p4)
Copy Number
log2 Ratio
Chr5
0.0e+00
5.0e+07
1.0e+08
1.5e+08

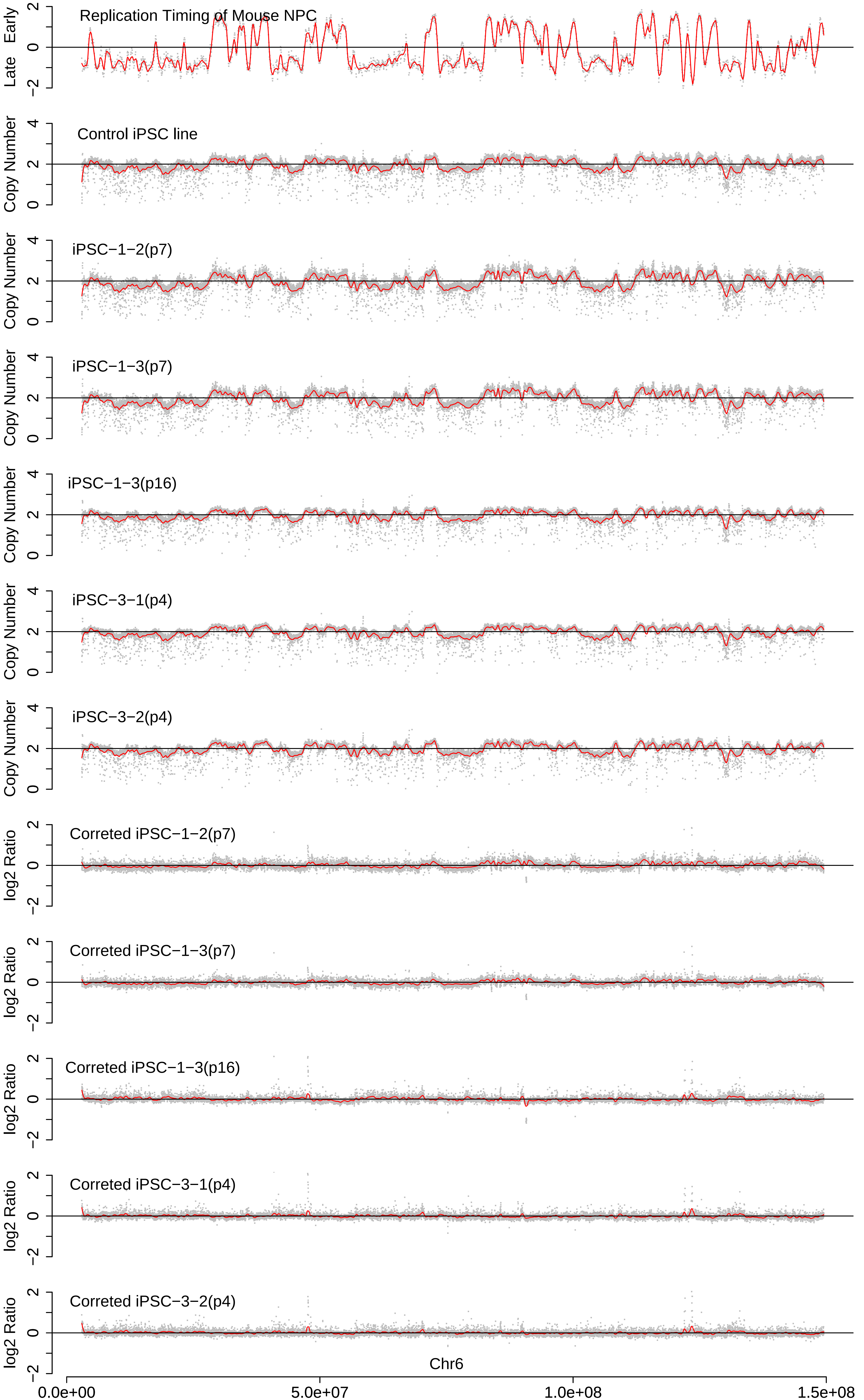

Replication Timing of Mouse NPC
Early
Late
Control iPSC line
iPSC−1−2(p7)
iPSC−1−3(p7)
iPSC−1−3(p16)
iPSC−3−1(p4)
iPSC−3−2(p4)
Copy Number
Correted iPSC−1−2(p7)
Correted iPSC−1−3(p7)
Correted iPSC−1−3(p16)
Correted iPSC−3−1(p4)
Correted iPSC−3−2(p4)
log2 Ratio
Chr6
0.0e+00
5.0e+07
1.0e+08
1.5e+08

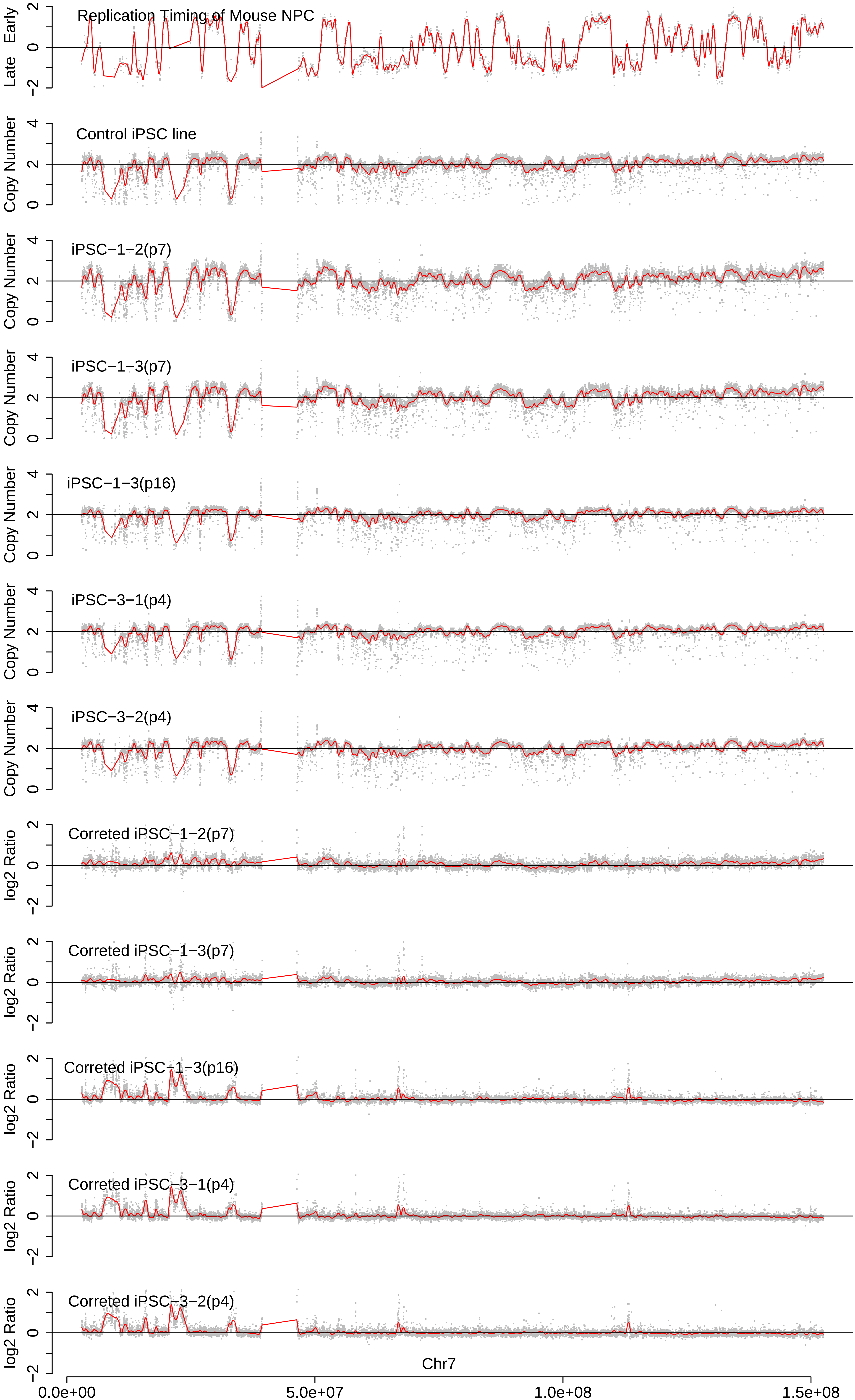

Replication Timing of Mouse NPC
Early
Late
Control iPSC line
iPSC−1−2(p7)
iPSC−1−3(p7)
iPSC−1−3(p16)
iPSC−3−1(p4)
iPSC−3−2(p4)
Copy Number
Correted iPSC−1−2(p7)
Correted iPSC−1−3(p7)
Correted iPSC−1−3(p16)
Correted iPSC−3−1(p4)
Correted iPSC−3−2(p4)
log2 Ratio
Chr7
0.0e+00
5.0e+07
1.0e+08
1.5e+08

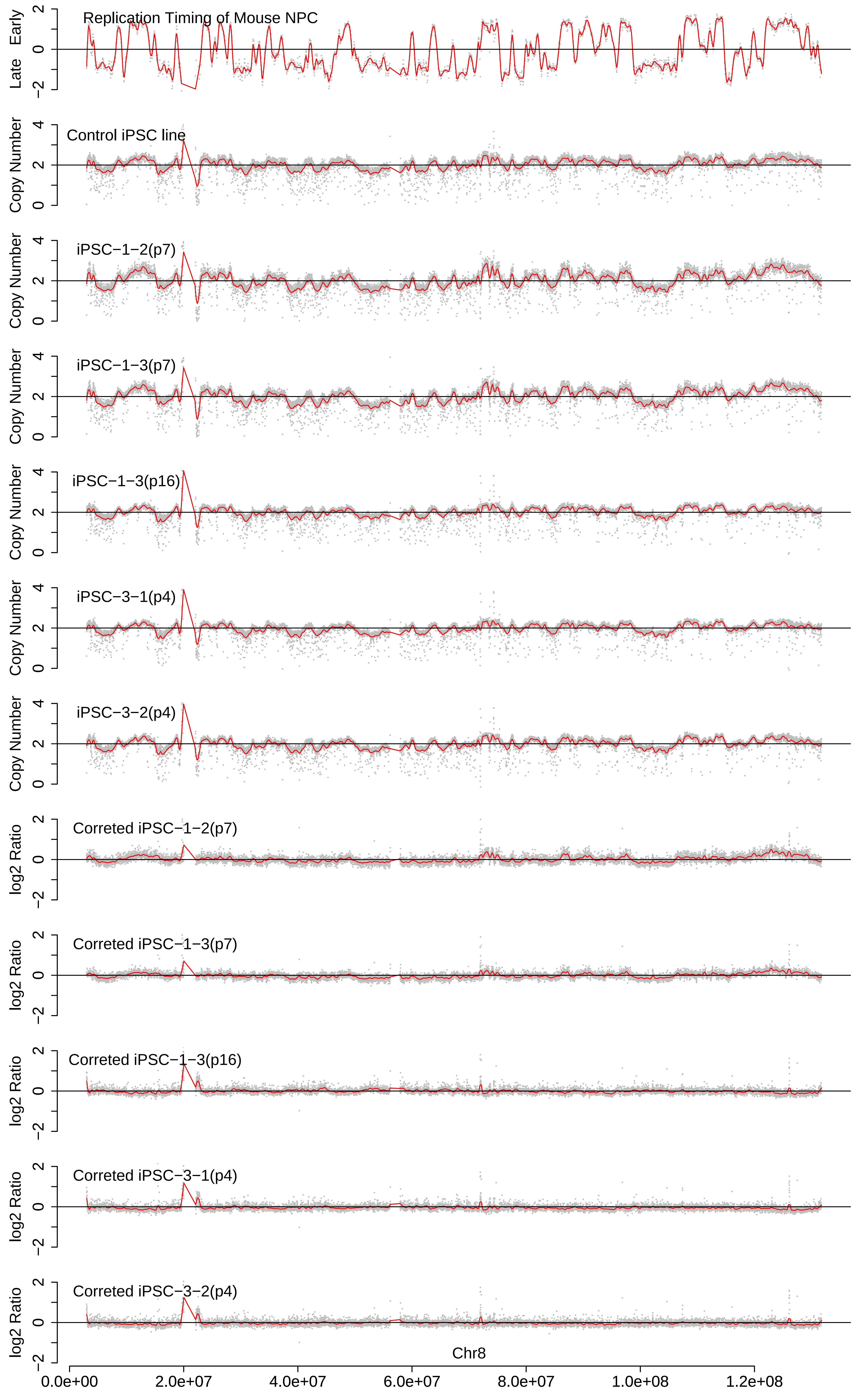
Replication Timing of Mouse NPC
Early
Late
Control iPSC line
iPSC−1−2(p7)
iPSC−1−3(p7)
iPSC−1−3(p16)
iPSC−3−1(p4)
iPSC−3−2(p4)
Copy Number
Correted iPSC−1−2(p7)
Correted iPSC−1−3(p7)
Correted iPSC−1−3(p16)
Correted iPSC−3−1(p4)
Correted iPSC−3−2(p4)
log2 Ratio
Chr8
0.0e+00
2.0e+07
4.0e+07
6.0e+07
8.0e+07
1.0e+08
1.2e+08

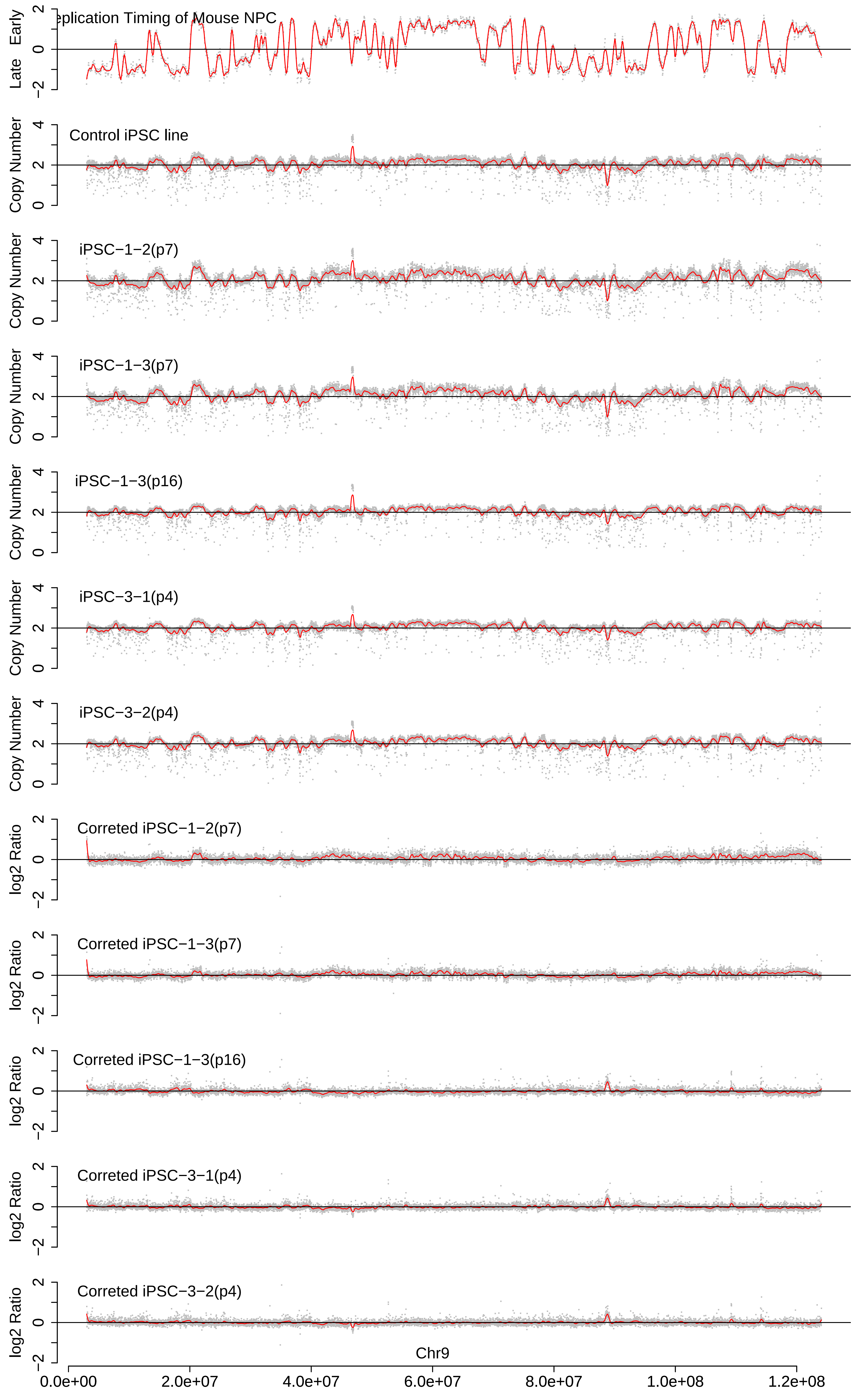

eplication Timing of Mouse NPC
Early
Late
Control iPSC line
iPSC−1−2(p7)
iPSC−1−3(p7)
iPSC−1−3(p16)
iPSC−3−1(p4)
iPSC−3−2(p4)
Copy Number
Correted iPSC−1−2(p7)
Correted iPSC−1−3(p7)
Correted iPSC−1−3(p16)
Correted iPSC−3−1(p4)
Correted iPSC−3−2(p4)
log2 Ratio
Chr9
0.0e+00
2.0e+07
4.0e+07
6.0e+07
8.0e+07
1.0e+08
1.2e+08

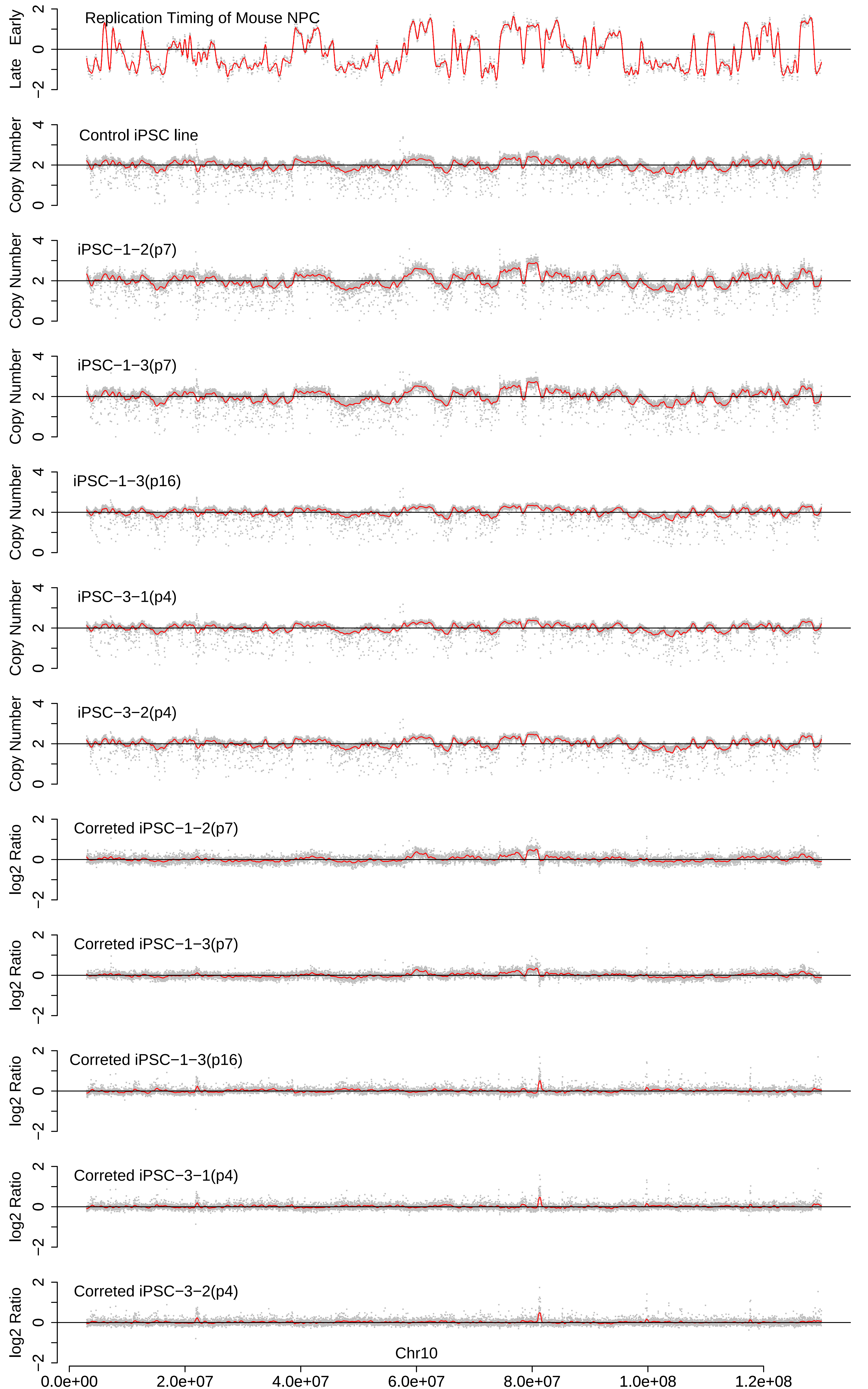

Replication Timing of Mouse NPC
Early
Late
Control iPSC line
iPSC−1−2(p7)
iPSC−1−3(p7)
iPSC−1−3(p16)
iPSC−3−1(p4)
iPSC−3−2(p4)
Correted iPSC−1−2(p7)
Correted iPSC−1−3(p7)
Correted iPSC−1−3(p16)
Correted iPSC−3−1(p4)
Correted iPSC−3−2(p4)
Copy Number
log2 Ratio
Chr10
0.0e+00
2.0e+07
4.0e+07
6.0e+07
8.0e+07
1.0e+08
1.2e+08

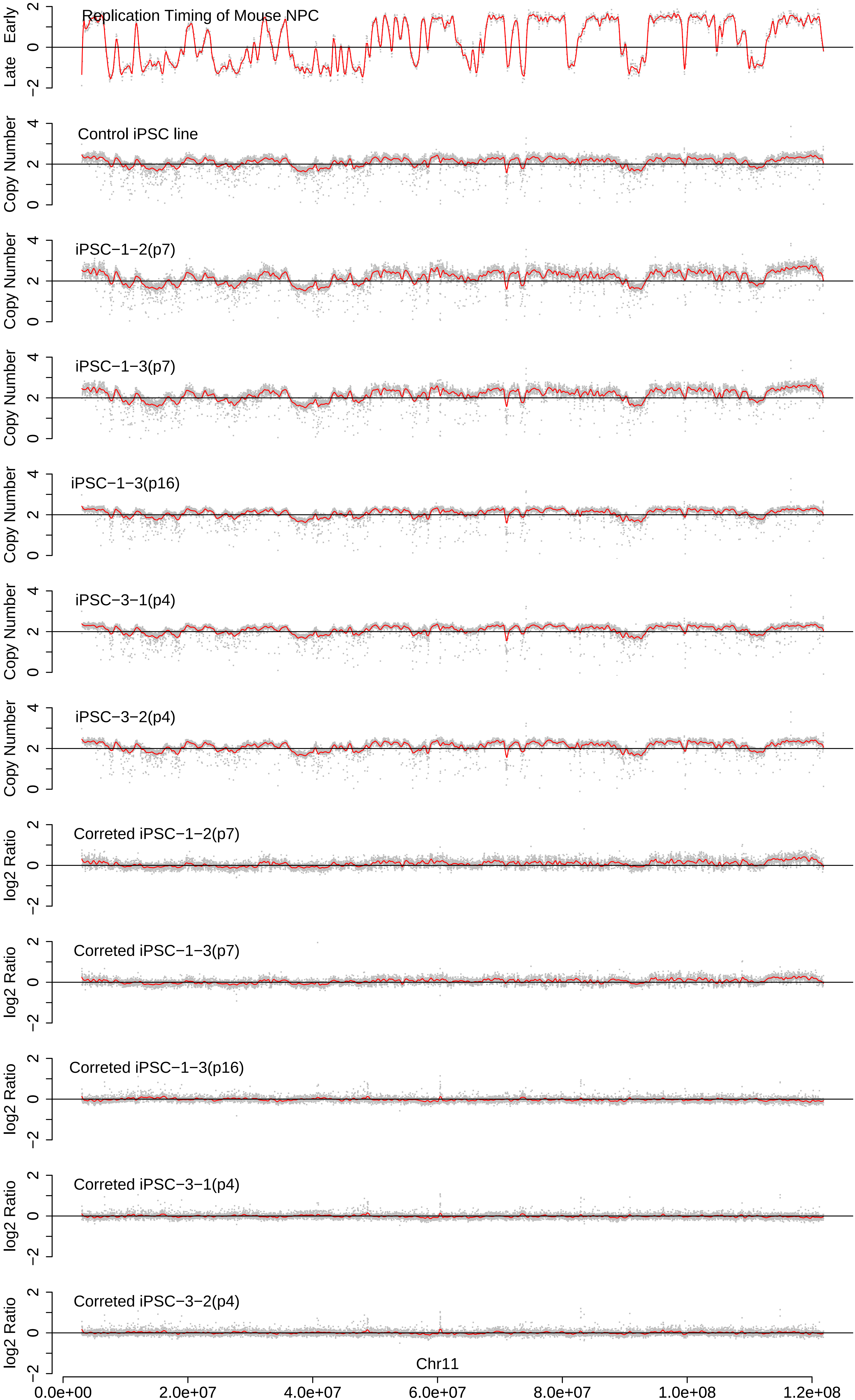
Replication Timing of Mouse NPC
Early
Late
Control iPSC line
iPSC−1−2(p7)
iPSC−1−3(p7)
iPSC−1−3(p16)
iPSC−3−1(p4)
iPSC−3−2(p4)
Copy Number
Correted iPSC−1−2(p7)
Correted iPSC−1−3(p7)
Correted iPSC−1−3(p16)
Correted iPSC−3−1(p4)
Correted iPSC−3−2(p4)
log2 Ratio
Chr11
0.0e+00
2.0e+07
4.0e+07
6.0e+07
8.0e+07
1.0e+08
1.2e+08

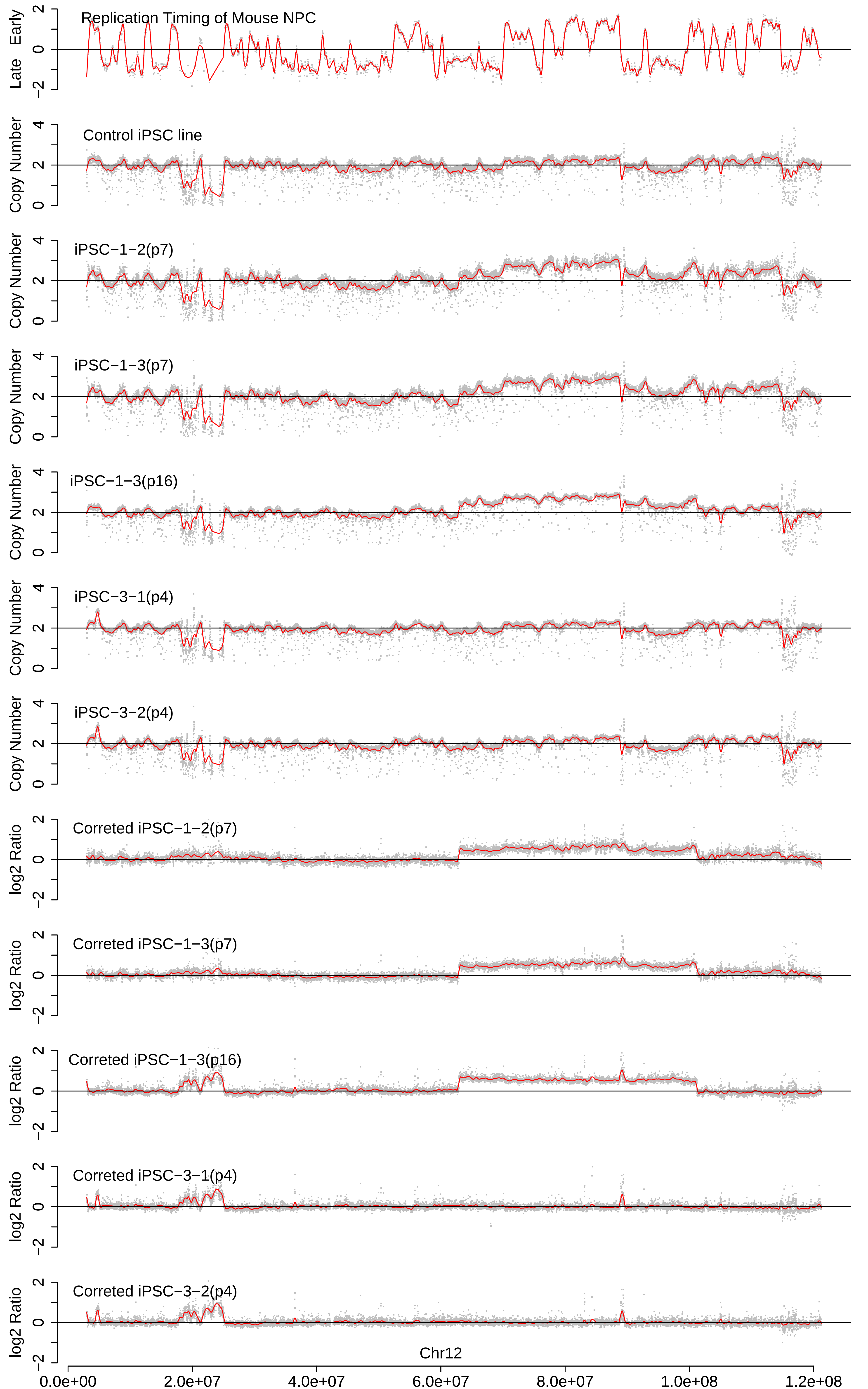

Replication Timing of Mouse NPC
Early
Late
Control iPSC line
iPSC−1−2(p7)
iPSC−1−3(p7)
iPSC−1−3(p16)
iPSC−3−1(p4)
iPSC−3−2(p4)
Correted iPSC−1−2(p7)
Correted iPSC−1−3(p7)
Correted iPSC−1−3(p16)
Correted iPSC−3−1(p4)
Correted iPSC−3−2(p4)
Copy Number
log2 Ratio
Chr12
0.0e+00
2.0e+07
4.0e+07
6.0e+07
8.0e+07
1.0e+08
1.2e+08

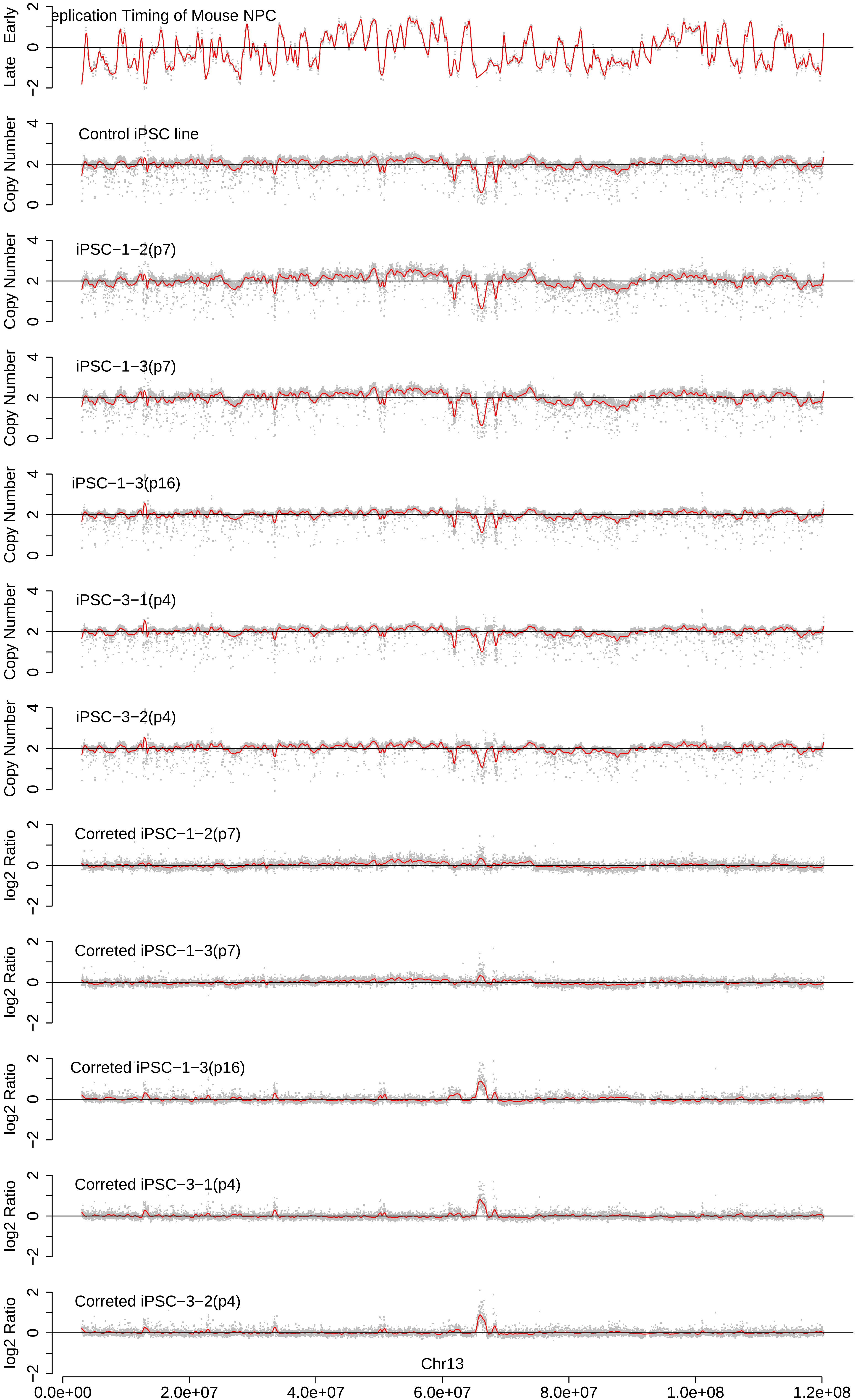

eplication Timing of Mouse NPC
Early
Late
Control iPSC line
iPSC−1−2(p7)
iPSC−1−3(p7)
iPSC−1−3(p16)
iPSC−3−1(p4)
iPSC−3−2(p4)
Correted iPSC−1−2(p7)
Correted iPSC−1−3(p7)
Correted iPSC−1−3(p16)
Correted iPSC−3−1(p4)
Correted iPSC−3−2(p4)
Copy Number
log2 Ratio
Chr13
0.0e+00
2.0e+07
4.0e+07
6.0e+07
8.0e+07
1.0e+08
1.2e+08

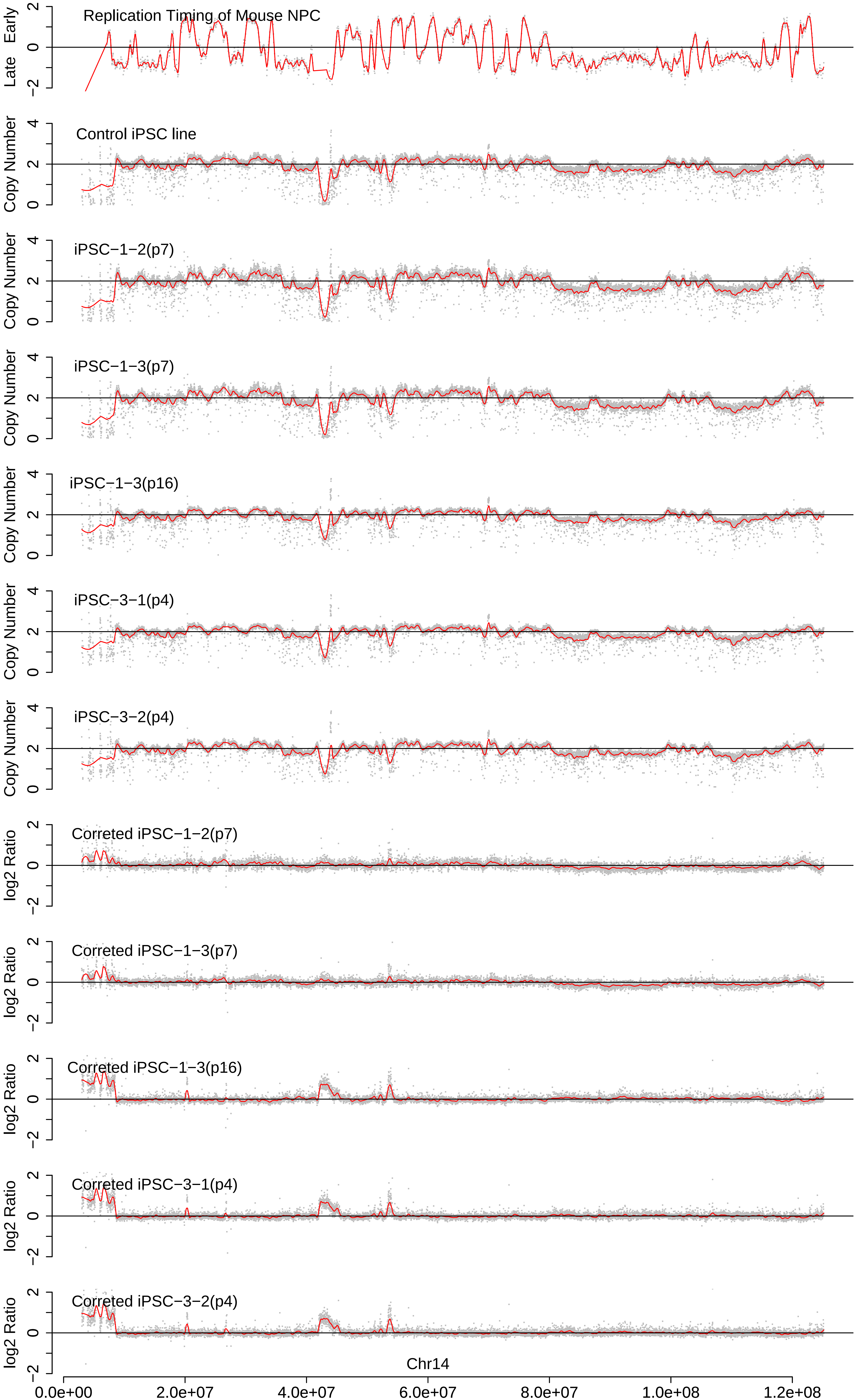
Replication Timing of Mouse NPC
Early
Late
Control iPSC line
iPSC−1−2(p7)
iPSC−1−3(p7)
iPSC−1−3(p16)
iPSC−3−1(p4)
iPSC−3−2(p4)
Correted iPSC−1−2(p7)
Correted iPSC−1−3(p7)
Correted iPSC−1−3(p16)
Correted iPSC−3−1(p4)
Correted iPSC−3−2(p4)
Copy Number
log2 Ratio
Chr14
0.0e+00
2.0e+07
4.0e+07
6.0e+07
8.0e+07
1.0e+08
1.2e+08

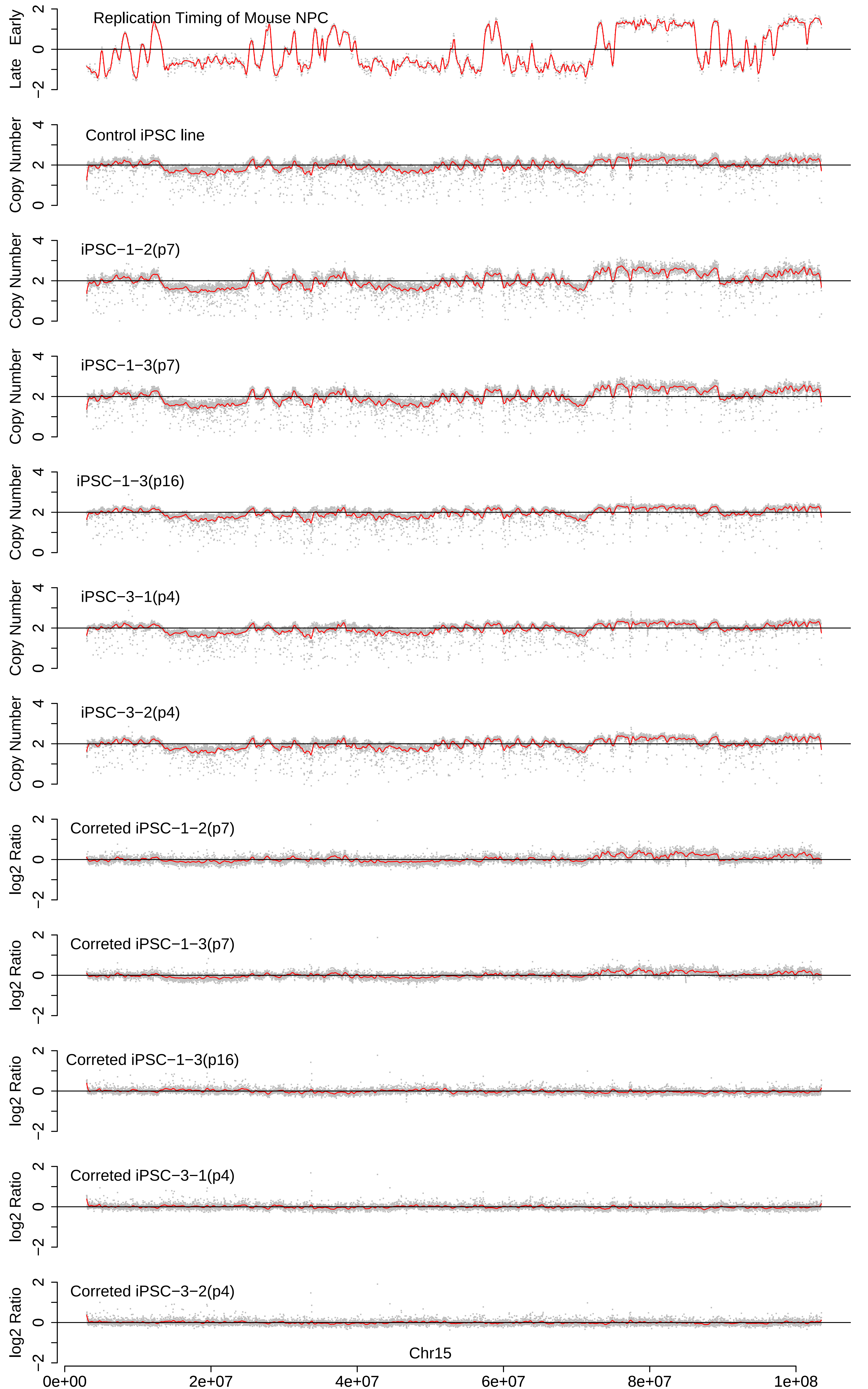

Replication Timing of Mouse NPC
Early
Late
Control iPSC line
iPSC−1−2(p7)
iPSC−1−3(p7)
iPSC−1−3(p16)
iPSC−3−1(p4)
iPSC−3−2(p4)
Copy Number
Correted iPSC−1−2(p7)
Correted iPSC−1−3(p7)
Correted iPSC−1−3(p16)
Correted iPSC−3−1(p4)
Correted iPSC−3−2(p4)
log2 Ratio
Chr15
0e+00
2e+07
4e+07
6e+07
8e+07
1e+08

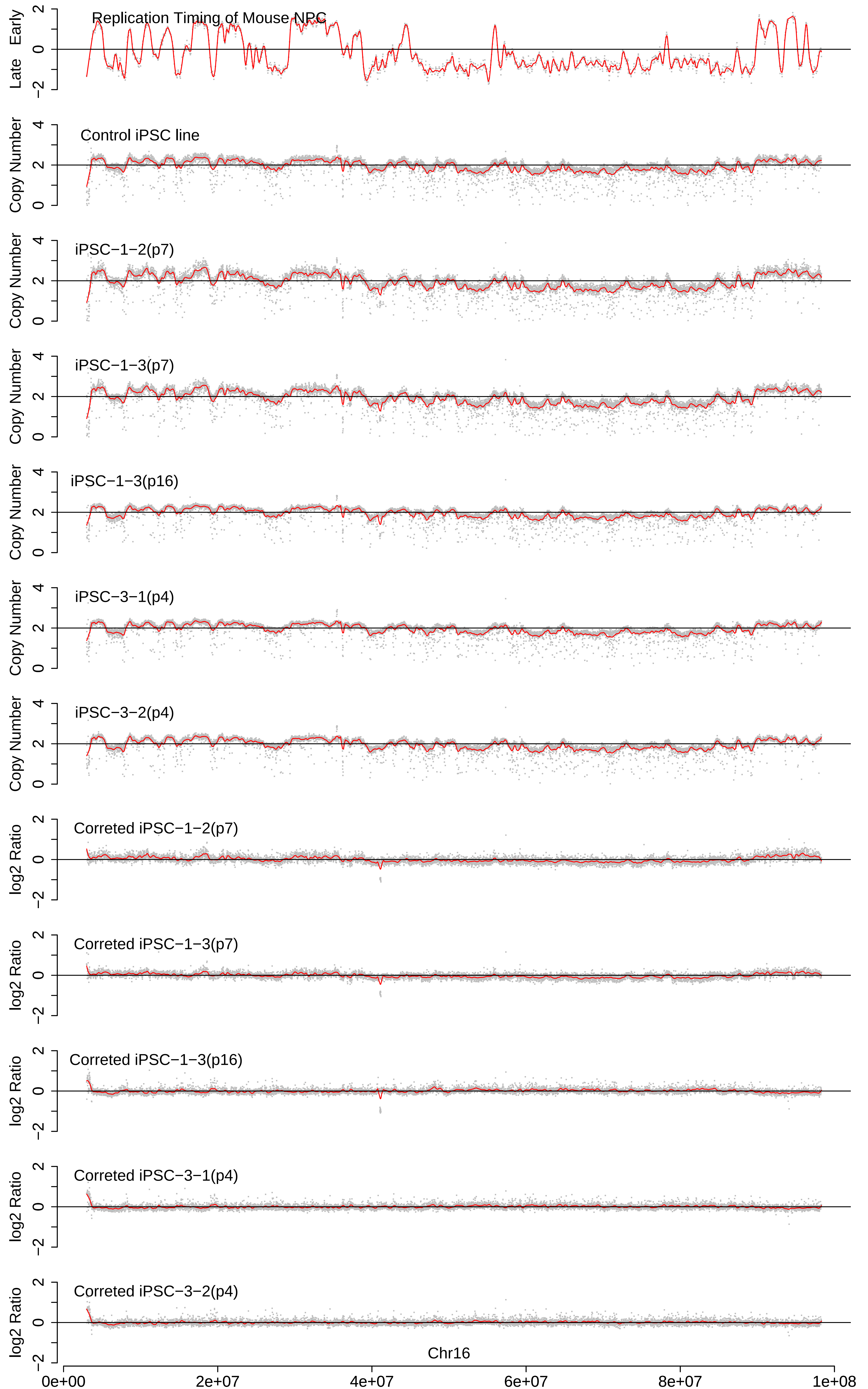

Replication Timing of Mouse NPC
Early
Late
Control iPSC line
iPSC−1−2(p7)
iPSC−1−3(p7)
iPSC−1−3(p16)
iPSC−3−1(p4)
iPSC−3−2(p4)
Copy Number
Correted iPSC−1−2(p7)
Correted iPSC−1−3(p7)
Correted iPSC−1−3(p16)
Correted iPSC−3−1(p4)
Correted iPSC−3−2(p4)
log2 Ratio
Chr16
0e+00
2e+07
4e+07
6e+07
8e+07
1e+08

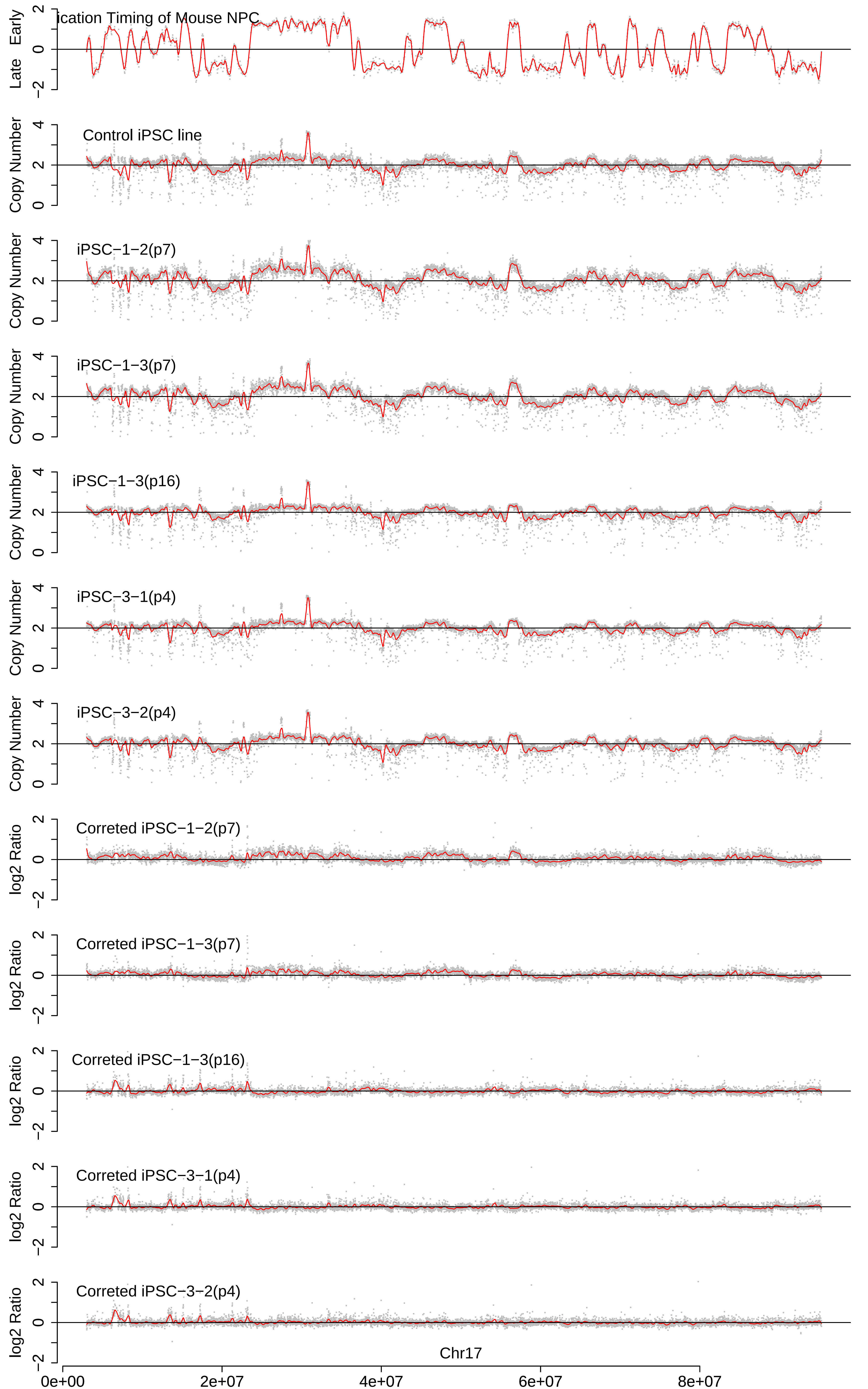

ication Timing of Mouse NPC
Early
Late
Control iPSC line
iPSC−1−2(p7)
iPSC−1−3(p7)
iPSC−1−3(p16)
iPSC−3−1(p4)
iPSC−3−2(p4)
Corrected iPSC−1−2(p7)
Corrected iPSC−1−3(p7)
Corrected iPSC−1−3(p16)
Corrected iPSC−3−1(p4)
Corrected iPSC−3−2(p4)
Copy Number
log2 Ratio
Chr17
0e+00
2e+07
4e+07
6e+07
8e+07

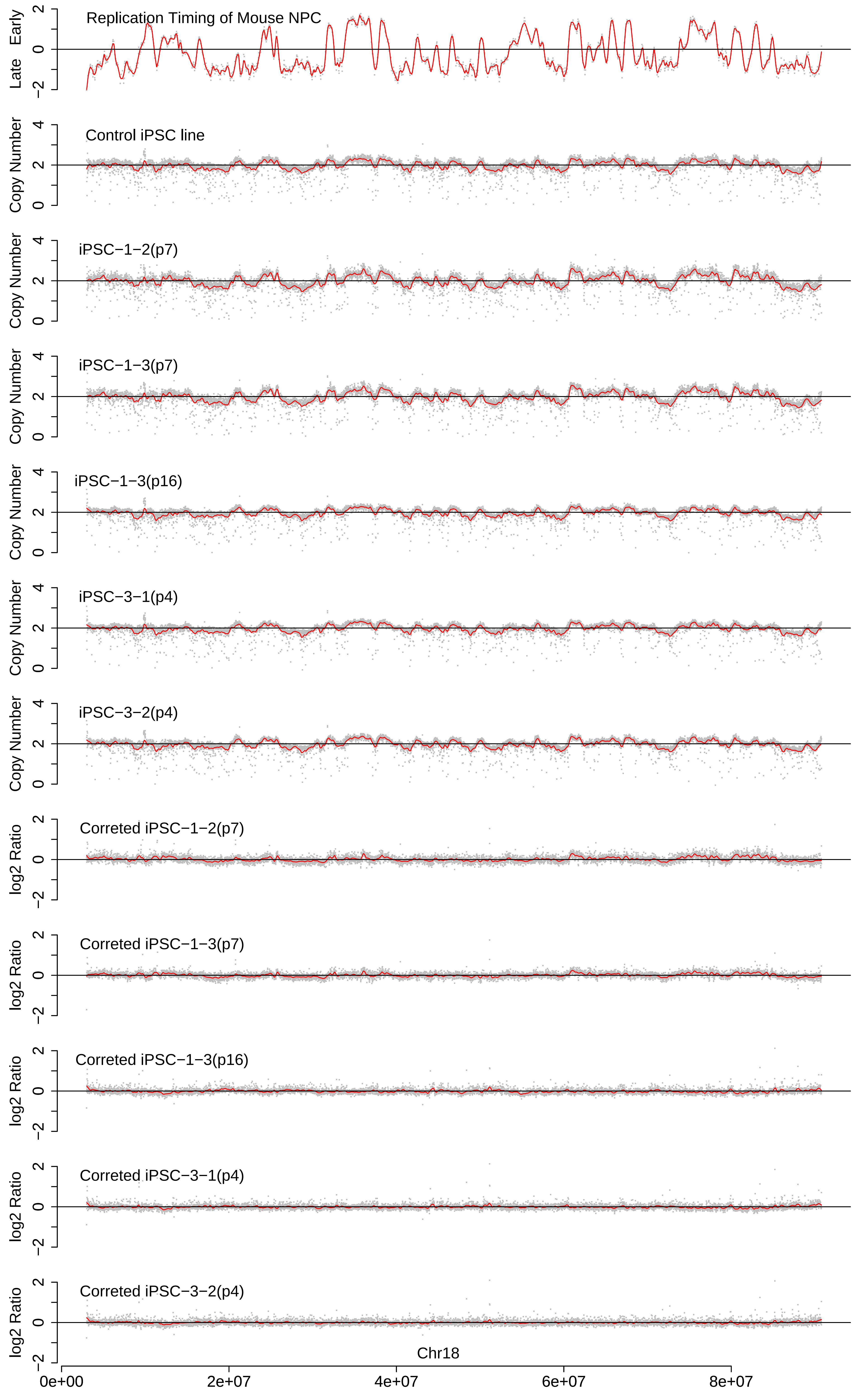
Replication Timing of Mouse NPC
Early
Late
Control iPSC line
iPSC−1−2(p7)
iPSC−1−3(p7)
iPSC−1−3(p16)
iPSC−3−1(p4)
iPSC−3−2(p4)
Copy Number
Correted iPSC−1−2(p7)
Correted iPSC−1−3(p7)
Correted iPSC−1−3(p16)
Correted iPSC−3−1(p4)
Correted iPSC−3−2(p4)
log2 Ratio
Chr18
0e+00
2e+07
4e+07
6e+07
8e+07

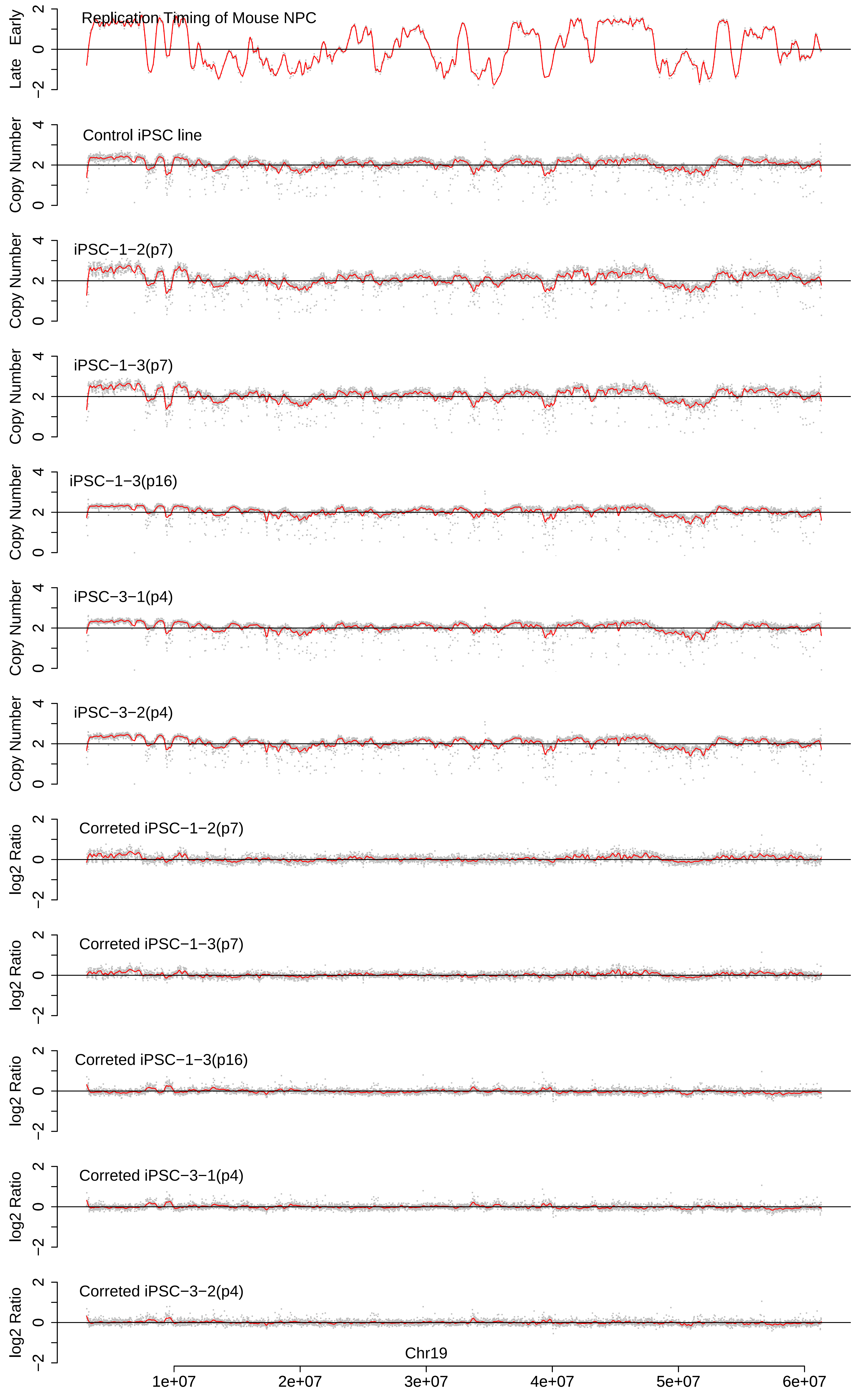
Replication Timing of Mouse NPC
Early
Late
Control iPSC line
iPSC−1−2(p7)
iPSC−1−3(p7)
iPSC−1−3(p16)
iPSC−3−1(p4)
iPSC−3−2(p4)
Correted iPSC−1−2(p7)
Correted iPSC−1−3(p7)
Correted iPSC−1−3(p16)
Correted iPSC−3−1(p4)
Correted iPSC−3−2(p4)
Copy Number
log2 Ratio
Chr19
1e+07
2e+07
3e+07
4e+07
5e+07
6e+07

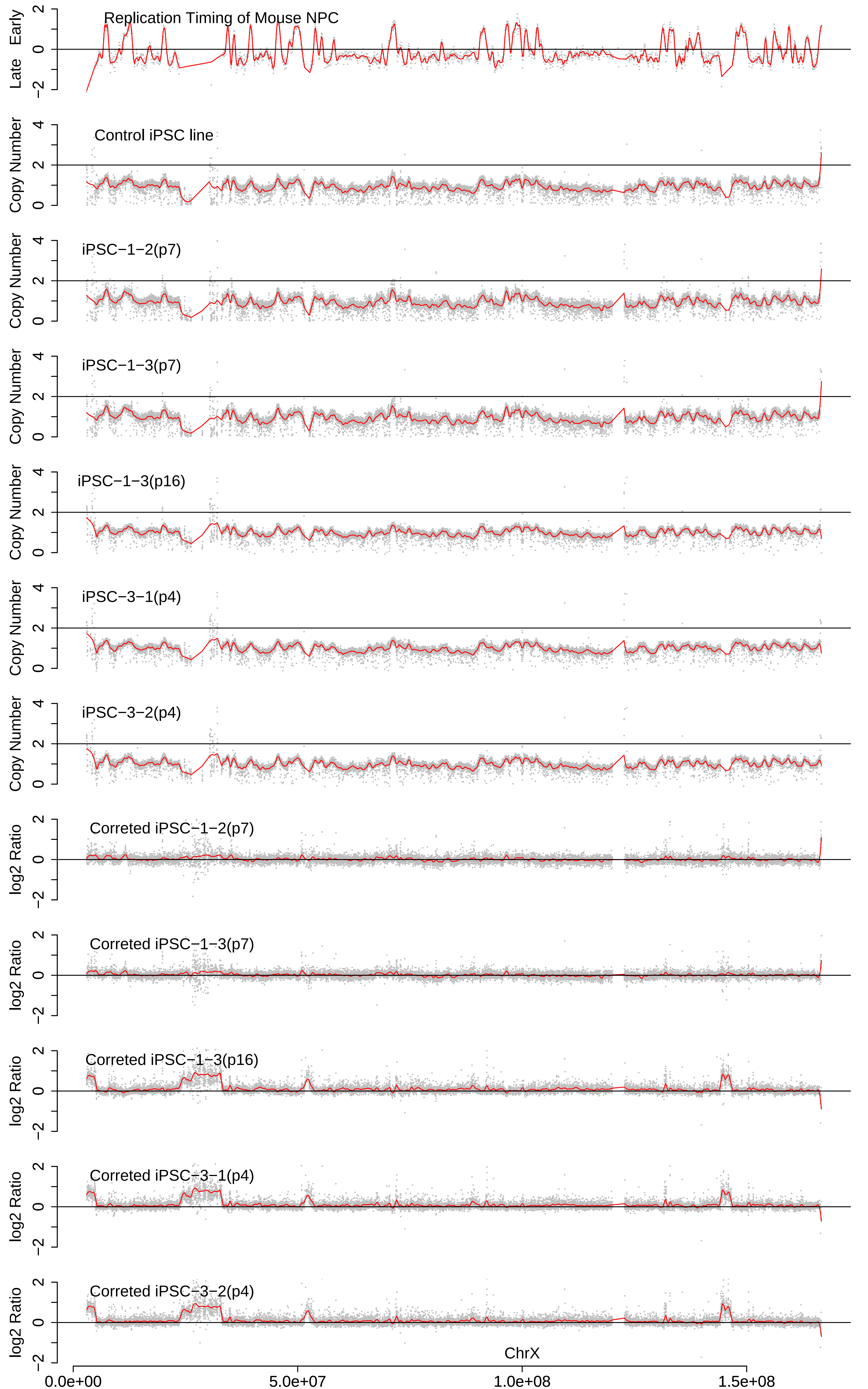

Replication Timing of Mouse NPC
Early
Late
2
0
−2
Control iPSC line
iPSC−1−2(p7)
iPSC−1−3(p7)
iPSC−1−3(p16)
iPSC−3−1(p4)
iPSC−3−2(p4)
Copy Number
4
2
0
Correted iPSC−1−2(p7)
Correted iPSC−1−3(p7)
Correted iPSC−1−3(p16)
Correted iPSC−3−1(p4)
Correted iPSC−3−2(p4)
log2 Ratio
ChrX
0.0e+00
5.0e+07
1.0e+08
1.5e+08
